# Exosite Binding in Thrombin: A Global Structural/Dynamic Overview of Complexes with Aptamers and Other Ligands

**DOI:** 10.3390/ijms221910803

**Published:** 2021-10-06

**Authors:** Romualdo Troisi, Nicole Balasco, Ida Autiero, Luigi Vitagliano, Filomena Sica

**Affiliations:** 1 Department of Chemical Sciences, University of Naples Federico II, Complesso Universitario di Monte Sant’Angelo, Via Cintia, I-80126 Naples, Italy; romualdo.troisi@unina.it; 2 Institute of Biostructures and Bioimaging, CNR, I-80134 Naples, Italy; nicole.balasco@unina.it (N.B.); ida.autiero@gmail.com (I.A.); luigi.vitagliano@unina.it (L.V.); 3 Molecular Horizon Srl, I-06084 Bettona, Italy

**Keywords:** thrombin, aptamer, exosite, functional partner, natural inhibitor, synthetic compound, structure, dynamic, inter-exosite communication, allostery

## Abstract

Thrombin is the key enzyme of the entire hemostatic process since it is able to exert both procoagulant and anticoagulant functions; therefore, it represents an attractive target for the developments of biomolecules with therapeutic potential. Thrombin can perform its many functional activities because of its ability to recognize a wide variety of substrates, inhibitors, and cofactors. These molecules frequently are bound to positively charged regions on the surface of protein called exosites. In this review, we carried out extensive analyses of the structural determinants of thrombin partnerships by surveying literature data as well as the structural content of the Protein Data Bank (PDB). In particular, we used the information collected on functional, natural, and synthetic molecular ligands to define the anatomy of the exosites and to quantify the interface area between thrombin and exosite ligands. In this framework, we reviewed in detail the specificity of thrombin binding to aptamers, a class of compounds with intriguing pharmaceutical properties. Although these compounds anchor to protein using conservative patterns on its surface, the present analysis highlights some interesting peculiarities. Moreover, the impact of thrombin binding aptamers in the elucidation of the cross-talk between the two distant exosites is illustrated. Collectively, the data and the work here reviewed may provide insights into the design of novel thrombin inhibitors.

## 1. Introduction—Overview of Thrombin: A Multi-Partner Serine Protease Involved in Blood Coagulation

**Figure 1:**
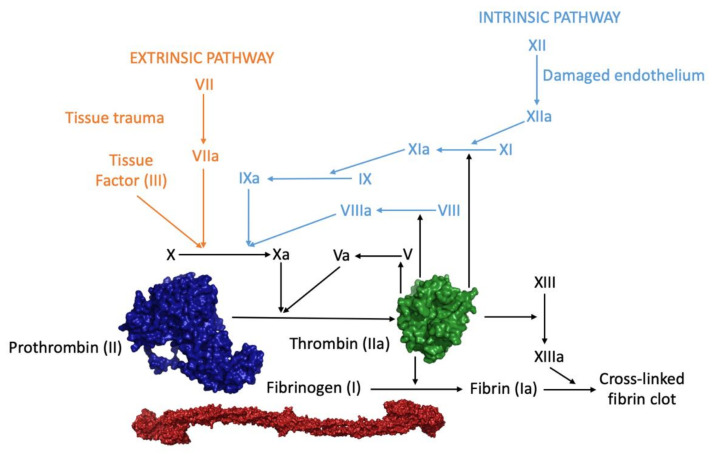
Blood coagulation cascade. The three-dimensional structures of prothrombin (PDB entry 6c2w), thrombin (PDB entry 1ppb), and its substrate (fibrinogen, PDB entry 3ghg) are shown.

α-Thrombin (coagulation factor IIa), hereafter referred to as thrombin, is the ultimate trypsin-like serine protease and is produced by the upstream activation of blood coagulation cascade [1] (Figure 1). Vascular injury triggers the aggregation of platelets, forming a plug at the damaged site (primary hemostasis). Fibrin activation is brought about by two convergent pathways: intrinsic and extrinsic (secondary hemostasis). The intrinsic pathway involves factors XII, XI, IX, and VIII and is activated through exposed endothelial collagen, while the extrinsic pathway involves factor VII and is caused by an external trauma. Both pathways convene in a final common pathway, in which factors X, V, II, and I take part, beginning with the activation of factor X in factor Xa that can form, upon Ca^2+^ binding, a complex with factor V converting prothrombin into thrombin [2,3].

Thrombin is the key enzyme of the entire hemostatic process and is able to exert both procoagulant and anticoagulant functions [4,5] (Table 1).

Procoagulant roles entail fibrin generation and platelets aggregation, while the anticoagulant action involves protein C activation. Moreover, thrombin promotes a massive amplification of the coagulation cascade by proteolytically converting other coagulation factors (V, VIII, and XI) to their active forms, eventually leading to its own generation, by a positive feedback mechanism [6]. The primary procoagulant effect of thrombin is the conversion of soluble fibrinogen into an insoluble fibrin network, which is further stabilized by factor XIIIa, which establishes covalent cross-linking connections on adjacent fibrin molecules [7]. Consequently, the red blood cells and platelets are trapped by the fibrin polymer, forming a stable plug that stops bleeding from the site of injury [8,9]. In addition, thrombin triggers platelets aggregation through the proteolysis of surface protease activated receptors (PARs), which are members of the G-protein-coupled receptors superfamily [10,11,12,13,14]. Thrombin binds to platelets through the surface glycoprotein GpIbα, which acts as a cofactor in PAR-1 cleavage but may also mediate platelet activation in a non-proteolytic manner [15]. In practice, thrombin is the orchestrator of blood coagulation regulation, which acts as a procoagulant agent when it converts fibrinogen into insoluble fibrin through a two-step cutting process and as anticoagulant agent when it binds to endothelial vascular cell proteins, called thrombomodulin [5,16]. This interaction leads both to the impediment of the substrate binding and to the activation of the anticoagulant protein C, which causes the inactivation of some coagulation factors (V and VIII) and the consequent decrease in thrombin production [17,18]. The delicate balance between these two functions is essential in a normal physiological state as it prevents the formation of clots in undamaged blood vessels and triggers the coagulation cascade in the damaged ones [2]. Interestingly, thrombin also plays a role in the protection of fibrin clots from degradation as, when associated to thrombomodulin, it activates the thrombin activatable fibrinolysis inhibitor (TAFI) [19,20].

**Figure 2:**
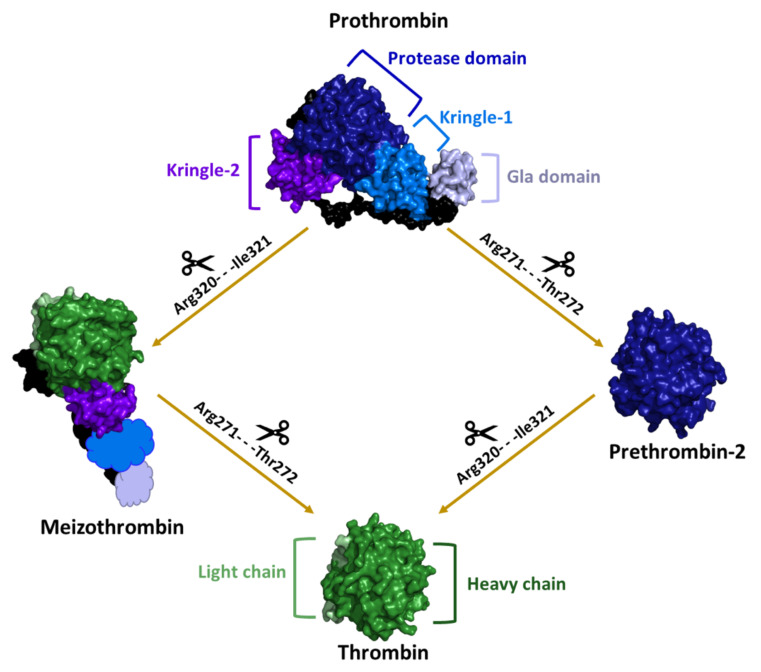
Schematic representation of the two possible pathways of thrombin generation from its precursor prothrombin. The three-dimensional structures of prothrombin (PDB entry 6c2w), thrombin (PDB entry 1ppb), meizothrombin (PDB entry 3e6p), and prethrombin-2 (PDB entry 4rn6) are shown. In the meizothrombin, the fragment 1 (GLA domain and kringle-1), which is absent in the crystal structure, is schematically represented. After the proteolysis of the Arg320-Ile321 peptide bond, the protease domain (dark blue) is divided into the heavy (dark green) and light (light green) chains, which are connected through a disulfide bond.

From the molecular point of view, active thrombin is a protein composed of two covalently linked polypeptide chains [1,4]. It is generated from the prothrombin precursor (Figure 1 and Figure 2), which is a single-chain glycoprotein with a molecular weight of about 72,000 Da that is produced in the liver and is co-translationally modified in a vitamin K-dependent reaction that converts 10–12 glutamic acids in the N terminus of the molecule to γ-carboxyglutamic acid (Gla) [4,21]. Prothrombin consists of four structural domains (Figure 2): the Gla domain (1–46); the region containing the γ-carboxylated glutamic acid residues; two kringle domains (65–143 and 170–248), which are involved in protein-protein interactions; and the trypsin-like serine protease domain (285–579), which contains the enzyme active site [22,23,24]. After the initiation of the coagulation cascade, factor Xa sequentially cleaves two peptide bonds in prothrombin, i.e., Arg320-Ile321 bond, to generate meizothrombin and then Arg271-Thr272. An alternative intermediate product denoted as prethrombin-2 can also be generated depending on the order of peptide bond cleavage and in the absence of a phospholipid membrane [25,26,27]. The proteolytically active thrombin molecule is made of a light chain, which comprises 36 residues, and a heavy chain, which is formed by 259 residue and contains the catalytic triad, held together by a single disulfide bond [28] (Figure 2).

**Figure 3:**
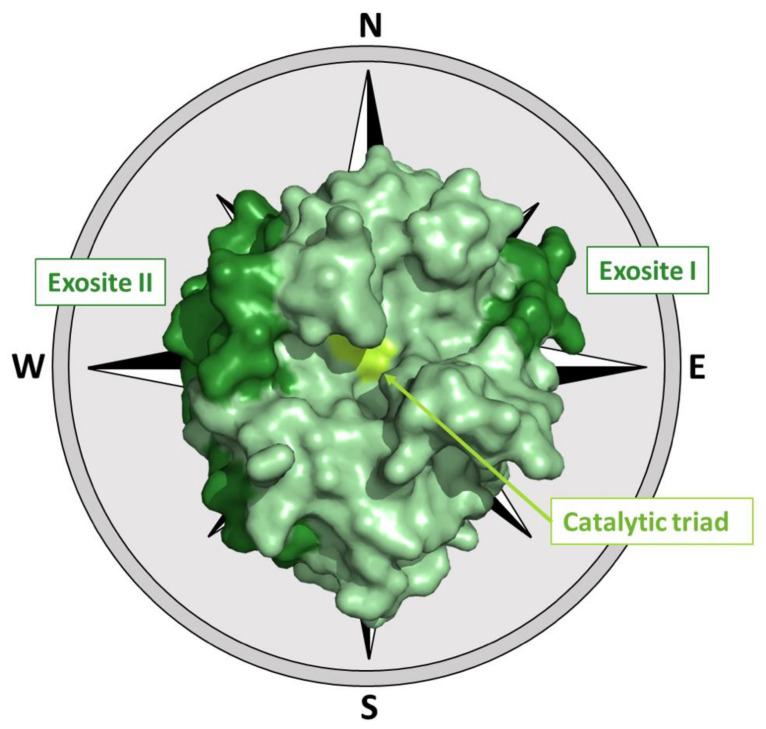
The three-dimensional structure of thrombin (PDB entry 1ppb). The relative locations of the active site and of the two exosites (Table S1) have been also shown using the cardinal points notation frequently adopted for thrombin [29,30].

Although thrombin fold closely resembles that of trypsin-like proteases, the distribution of the charged residues on its surface is somehow peculiar. The catalytic residues (His57, Asp102, and Ser195) are located at the edge of a negatively charged surface that is surrounded by two positive hot spots to the northeast and west of the active site, called exosites [29] (Figure 3 and Table S1).

The northeastern positive region (exosite I), which is located at about 10–15 Å from the base of the active site cleft and mostly comprises residues of the Arg67-Ile82 loop, plays a role in the interaction with several biological partners such as fibrinogen [31,32,33], fibrin [32,34,35], thrombomodulin [36,37,38,39], and thrombin receptor [40,41,42].

The patch that extends from the northwest to the southwest of the protein surface (exosite II) (Figure 3), which is a high positively charged and discontinuous region also located in the proximity of the C-terminal helix of the heavy chain, binds heparin and interacts with the kringle-2 domain in prothrombin [43,44,45,46]. The thrombin binding to heparin facilitates the formation of complexes with the physiological inhibitors, heparin cofactor II and antithrombin III [45,47].

Thrombin can perform the above-mentioned and other functions because of its ability to recognize a wide variety of substrates, inhibitors, and cofactors [4,45] (Table 1). Its structure is strongly modulated even by small ligands, including the Na^+^ ion. Indeed, the binding of the endogenous sodium to the protein induces a conformational transition from the so-called “slow form” of thrombin, which is considered to act as an anticoagulant for its propensity to bind thrombomodulin, to the “fast form” [48,49]. This allosteric transition leads to an improvement of the protein affinity for the substrate. Since thrombin is involved in a variety of biochemical pathways and has many substrates and partners [50,51] (Table 1), a remarkable number of strategies have been developed to modulate the activity of this protein. They can be differentiated on the basis of the protein region that is targeted, the catalytic site and/or its surface hot spots deputed to the interactions with the biological partners, or on the basis of the chemical nature of the inhibitors.

The development of thrombin modulators has been frequently inspired by either endogenous or exogenous natural inhibitors. Indeed, thrombin catalytic activity can be efficiently hampered by some endogenous serine protease inhibitors (serpin superfamily) such as antithrombin III (ATIII), heparin cofactor II (HCII), and protease nexin I (PNI) [46,52,53]. On the other hand, several potent exogenous inhibitors have been isolated from hematophagous organisms (Table 2), including hirudin and hemadin from the leeches *Hirudo medicinalis/Hirudinaria manillensis* (hirudin) and *Haemadipsa sylvestris* (hemadin) [29,54].

In this scenario, crystallographic studies on thrombin and on its complexes have been fundamental for highlighting the intrinsic structural properties of this enzyme, for unravelling the mechanism of action of its modulators, and for providing insights into the design and development of new classes of synthetic inhibitors (Table 2 and Table 3).

**Figure 4:**
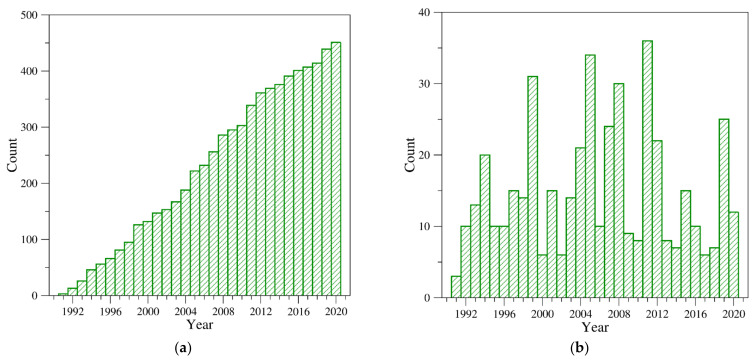
PDB entries containing at least one α-thrombin chain: (**a**) the cumulative temporal growth of these structures and (**b**) the number of structures deposited *per* year. Structures were selected by considering all PDB entries released within 2020 containing at least one chain with a sequence identity higher than 75% when compared with α-thrombin (Uniprot code: P00734). Considering the length of the thrombin (295 residues), in order to exclude protein fragments and precursors, the ensemble content was further refined by considering polypeptide chains with a number of residues comprised between 200 and 300. This survey yielded a structural ensemble of 451 structures from the following species: *Homo sapiens* (421 structures), *Bos Taurus* (23), and *Mus Musculus* (7).

Over the years, a number of insightful reviews focusing on specific aspects of thrombin structure have been published. Among others, these include (a) general descriptions of the structure of thrombin and its precursors [4,5,49,90,91,92,93,94,95,96,97,98,99,100], (b) the binding modes of active site inhibitors and/or functional partners [4,5,49,90,91,93,94,95,96,97,98,99,101,102,103], (c) the interaction with natural and/or allosteric inhibitors [4,5,49,90,91,93,94,95,96,97,98,99,101,102,103,104,105], and (d) the role of Na^+^ in thrombin conformational switch [1,4,5,49,93,94,95,96,98,99,100,103,104]. However, despite the ongoing interest in thrombin structural biology, evidenced by the continuous growth of three-dimensional structures deposited in the Protein Data Bank (PDB) (Figure 4), the progress achieved through structural characterizations of this protein in the last decade has not been reviewed.

This is somewhat surprising considering the remarkable results recently achieved in the definition of the long-distance and dynamic allosteric regulation of this protein [106,107], a topic in which the experimental and computational characterizations of thrombin complexes with aptamers, either single-stranded DNA or RNA molecules, which selectively bind to a specific target, have played a major role [108,109,110,111,112]. To fill this gap and to attain an up-to-date view of thrombin structural biology, we couple a survey of literature papers with an extensive analysis of PDB content. After a chronological and synthetic description of the milestones achieved in thrombin structural characterization, we illustrate the anatomy of the exosites and the variety of the binding modes of different classes of molecular ligands (peptides/proteins, nucleic acids, and heparin-like compounds), with particular attention paid to thrombin-aptamer recognition. Finally, by also considering computational studies, we provide an extensive description of the dynamic allostery recently highlighted by the study of thrombin-aptamer complexes.

## 2. Structural Characterization of Thrombin: A Chronological Perspective

As anticipated above to achieve a comprehensive and updated view of thrombin structural biology, we interrogated the PDB and selected 451 entries (Table S2) containing at least one thrombin chain (see the legend of Figure 4 for details). The inspection of the chronological distribution of these structures (Figure 4) indicates that starting from early 1990s, there has been continuous growth of the deposited structures.

Although thrombin is a relatively “old” protein from the structural perspective, it is worth noting that thrombin structures have been deposited in recent years at a remarkable pace. Indeed, since 2011 as many as 120 structures have been deposited, with a peak of 25 structures released in 2019. The first thrombin crystal structure, which was published in 1989 [113] and deposited in the PDB in 1991 (PDB entry 1ppb), contained a D-Phe-Pro-Arg chloromethylketone in the active site to prevent the auto-proteolysis of the protein and highlighted the analogies and the differences with the other trypsin-like proteinases. This study was soon followed by the first characterization of thrombin complexes with hirudin (PDB entry 3htc) [62], a peptide ligand of natural origin (Table 2). Within very few years from the initial structural characterization of thrombin, structures of complexes with rather large partners were determined. These studies unraveled the interaction modes of this protein with the kringle-2 domain of prothrombin [43]; with functional partners (thrombomodulin [114], heparin [115,116], platelet glycoprotein Ibα [117,118], and fibrinogen [119,120]); with the natural inhibitors rhodniin [72], ornithodorin [70], triabin [30], hemadin [61], staphylocoagulase [73]; and with aptamers (initially TBA [121] and, then, Toggle-25t [87]) (Table 2 and Table 3). In particular, TBA is the first DNA aptamer selected against thrombin [122]. It adopts an antiparallel G-quadruplex structure and recognizes the exosite I [80,121]. Conversely, Toggle-25t is an RNA aptamer that contains 2′-fluoropyrimidine nucleotides and binds thrombin exosite II [87].

In the last decade, structural studies on thrombin have provided information on thrombin modulation by natural inhibitors (anophelin [56], avathrin [57], cE5 salivary protein [55], IgA fab antithrombin antibody [67], madanin-1 [68], tsetse thrombin inhibitor [78], and variegin [79]) derived from different organisms or bivalirudin, an artificial anticoagulant peptide, and mimic of hirudin [58]. However, although many of the recent structural studies on thrombin represent variations on the theme of the active site inhibition by different compounds in the presence of hirugen at exosite I, the most important contributions achieved in the last decade are related to the analysis of aptamers targeting thrombin. Indeed, 18 out of a total of 22 thrombin-aptamer complexes reported in the PDB have been determined in the last decade [80,81,82,83,84,85,86,88,89,109,112].

The first structures (PDB entries 3qlp, 4dih, 4dii) unambiguously established the way in which the aptamers specific for exosite I interact with thrombin [80,81]. Indeed, the structures of the protein complexes with the antiparallel G-quadruplex TBA (Figure S1) or its mTBA variant definitely identify the TT loops as the driving structural feature for the binding of these aptamers to thrombin exosite I. In particular, these loops act as a pincer-like system that embraces the protruding region of exosite I. On the contrary, the remaining three-residue loop (TGT), placed on the other side of the G-quadruplex tetrads, is placed far from the exosite I region (Figure S1) and weakly interacts with different areas of symmetry-related thrombin molecules [80,81]. Furthermore, these crystal structures revealed the effects of the ionic species in the modulation of the thrombin-TBA recognition. Indeed, the alkaline ions (Na^+^ or K^+^), which also influence the inhibitory activity of TBA, affect the flexibility of the aptamer by inducing subtle perturbations of a few key interactions at the protein–aptamer interface [80].

Subsequently, the folding and the interaction surface of more sophisticated aptamers were investigated. The first crystal structure of the complex between thrombin and a duplex/quadruplex aptamer (PDB entry 4i7y) was published in 2013 [89]. In this structure, the peculiar folding of the HD22_27mer aptamer, which ensures a remarkably high interaction surface with thrombin exosite II involving both the aptamer structural domains, was revealed (Figure S2). In particular, HD22_27mer adopts a kinked conformation in which the helical axis of the regular duplex segment and that of a *pseudo*-G-quadruplex motif are approximately at right angle. Conversely, the subsequent structures (PDB entries 5cmx, 6evv, 6gn7) are related to the complexes between thrombin and duplex/quadruplex aptamers able to recognize exosite I (RE31 and NU172) [85,86]. These oligonucleotides, which display compact folding with the duplex region that stacks on the antiparallel G-quadruplex domain, interact with the protein exosite I only via the G-quadruplex domain. In particular, in thrombin-NU172 complexes, the involvement of the three-residue loop in the interaction with the protein exosite I was revealed for the first time (Figure S3). This loop is also directly involved in the stabilization of the duplex/quadruplex transition region, giving to the aptamer a compactness that contributes to its high anticoagulant activity [86].

More recently, research has been focused on the analysis of the effect of chemical modifications on the folding and interaction properties of Toggle-25t (PDB entry 5do4) [88] and TBA (PDB entries 4lz1, 4lz4, 6eo6, 6eo7, 6z8v, 6z8w, and 6z8x) [82,83,84]. In particular, the structures of the complexes between thrombin and the TBA mutants point out the tendency of the unmodified TT loop to interact with the region of exosite I (called A-region), engaging a high contact area. Consequently, the chemical modifications of the other TT loop modulate the binding affinity of the aptamer by increasing or decreasing the contacts of the oligonucleotide with the other exosite I region (called B-region), even affecting the aptamer flexibility [82,83,84]. The structure of the complex between thrombin and the Toggle-25t mutant (named AF113-18) reveals a localized induced-fit rearrangement of the modification-containing nucleotide, which contributes to the improvement of the interaction with the protein exosite II [88].

Finally, the aptamer-guided communication between the two thrombin exosites was investigated in three ternary complex structures in which the thrombin is sandwiched between HD22_27mer and TBA variants (PDB entries 5ew1, 5ew2) [109] or NU172 (PDB entry 7ntu) [112]. Some of these structures, which are embedded in different packing organizations, displayed subtle differences only in the conformation of HD22_27mer and in its interaction with the exosite II surface [109]. The presence in the protein active site of the covalently bound inhibitor PPACK, which is used in crystallization experiments to avoid the heterogeneity of protein solution induced by autoproteolysis, influences the intrinsic mobility of the protein, preventing the marked structural identification of an interplay between the two exosites [109,112].

## 3. Thrombin Recognition of Functional, Natural, and Synthetic Partners: Anatomy of Exosites

The variety and the importance of thrombin in the coagulation cascade have stimulated the generation of natural anticoagulant inhibitors by blood-sucking animals such as vampire bats, ticks, leeches, and hookworms [29,54]. Natural inhibitors whose interactions with thrombin have been structurally elucidated are reported in Table 2. Moreover, the pharmaceutical interest in the modulation of specific thrombin activities has also led to the generation of synthetic thrombin-interacting molecules with different chemical natures (Table 2 and Table 3).

**Figure 5:**
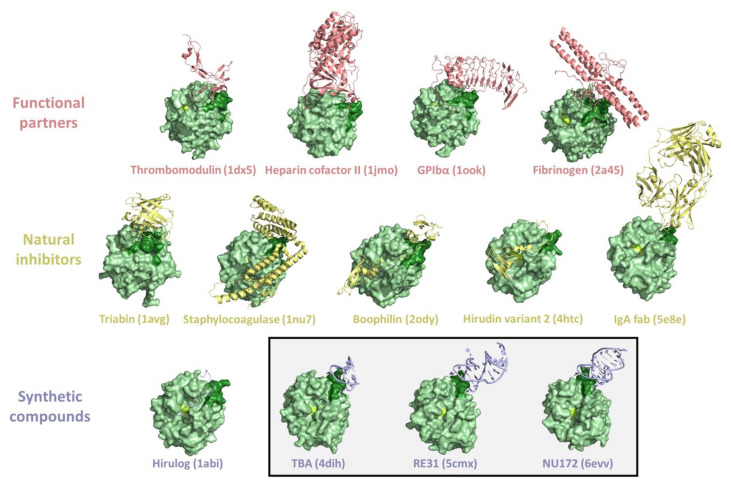
Representative examples of crystallographic structures of thrombin complexes with exosite I interactors (PDB entries are within brackets). The exosite I and the active triad of thrombin are in dark green and lemon, respectively. A box identifies the thrombin-aptamer complexes.

**Figure 6:**
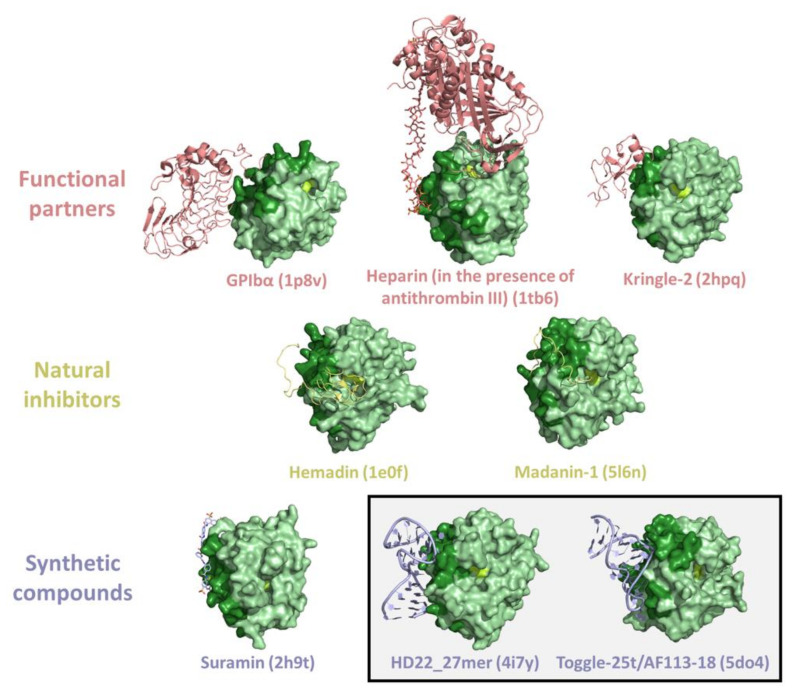
Representative examples of crystallographic structures of thrombin complexes with exosite II interactors (PDB entries are within brackets). The exosite II and the active triad of thrombin are in dark green and lemon, respectively. A box identifies the thrombin-aptamer complexes.

The involvement of thrombin in multiple partnerships can be accomplished by the presence of different interacting-prone regions on the protein surface. As previously stated, in addition to the active site, where substrates and some inhibitors bind, thrombin surface is characterized by two hot spot regions (exosite I and II) that are exploited for partner recognition [1] (Figure 3). Obviously, the same regions are the target of both natural and synthetic inhibitors/modulators. The large amount of structural data accumulated over the years provides a detailed view of these interacting-prone regions (Figure 5 and Figure 6).

The exosites have been effectively exploited by thrombin inhibitors of animal or synthetic origin. In particular, structural information has been obtained for the exosite I binding of natural ligands, such as hirudin [62,123,124], avathrin [57], staphylocoagulase [73], triabin [30], anophelin [56], boophilin [59], ornithodorin [70], rhodniin [72], cE5 salivary protein [55], IgA fab antithrombin antibody [67], and variegin [79], or of some synthetic compounds, such as bivalirudin [58], hirulog [63,125,126], hirunorm [65,127], hirutonin [66], and aptamers (TBA, RE31, NU172) [80,85,86] (Figure 5). Structural data have also been obtained for exosite II binding of both natural ligands (hemadin, madanin-1, and tsetse thrombin inhibitor) [61,68,78] and synthetic compounds (suramin and aptamers like Toggle-25t and HD22_27mer) [74,87,88,89] (Figure 6).

**Figure 7:**
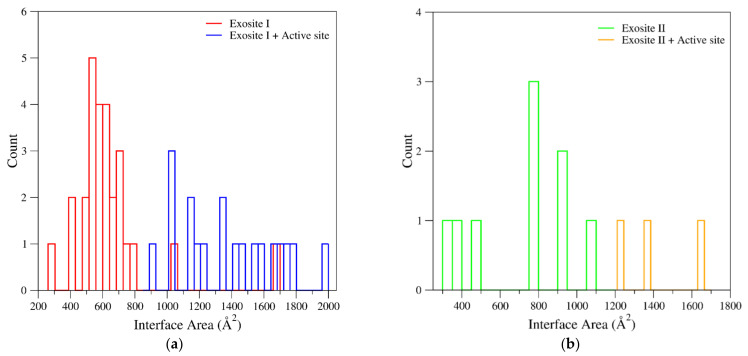
Distribution of the interface areas found in the thrombin-ligand complexes at (**a**) exosite I and (**b**) exosite II. In the panel (**a**), blue and red bars represent ligands bound either to both exosite I and active site or exclusively to the exosite. Similarly, in the panel (**b**), orange and green bars represent ligands bound either to both exosite II and active site or exclusively to the exosite. Structures of meizothrombin and prethrombin-2 were not considered. Only ligands corresponding to biomolecules (peptides/proteins, nucleic acids, and heparin-like compounds) bound to thrombin were analyzed. If multiple thrombin-ligand complexes were found in the PDB, the one refined at the highest resolution or the largest fragment was considered.

A global analysis of the complexes between thrombin and ligands provides interesting insights into the exosite anatomy and into the main forces that stabilize thrombin partnerships. Using the structures identified by the survey described in the previous paragraph, we selected thrombin complexes with biomolecules binding to exosites. From this analysis, we identified 46 non-redundant complexes in which the ligand was bound to the exosite I (see the legend of Figure 7 for the criteria used to select the non-redundant structures). Nineteen of these ligands simultaneously also bind the protein active site (exosite I + active site). We also selected 12 non-redundant complexes in which the exosite II is bound; three of these ligands also bind the active site (exosite II + active site). Structural characterizations of ternary complexes with the two thrombin exosites simultaneously bound to different ligands have been so far reported only for aptamers. These correspond to the structural characterizations made in our laboratory of thrombin bound to TBAΔT3/HD22_27mer (PDB entry 5ew1) [109], to TBAΔT12/HD22_27mer (PDB entry 5ew2) [109], or to NU172/HD22_27mer (PDB entry 7ntu) [112]. On these ensembles of thrombin-ligand complexes, for each structure we computed the interface area and the number of intermolecular hydrogen bonds/salt bridges with the PISA program [128] available online (https://www.ebi.ac.uk/pdbe/pisa/) using the default parameters and settings. Data for ligands bound at exosite I and exosite II are reported in Table 4 and Table 5, respectively.

As shown in Figure 7a and Table 4, the interface areas exhibited by the complexes of ligands bound to the exosite I are highly differentiated, spanning from ~300 to 1700 Å^2^. Obviously, largest thrombin-ligand interacting interfaces are found for ligands that simultaneously bind exosite I and active site. The inspection of the surface areas of the complexes with ligands exclusively bound to the exosite I indicates that most of them clustered in the 400–800 Å^2^ interval. There are, however, significant outliers. In particular, the minimal exosite I recognition region (297 Å^2^) is detected in the complex formed by thrombin with a peptide fragment of the functional partner Factor V (PDB entry 3p70) [129]. On the other hand, rather large interfaces (>1000 Å^2^) are found in complexes with exosite I-anchoring proteins (platelet glycoprotein Ibα and staphylocoagulase) [73,117]. In this framework, aptamers present rather typical and generally constant interface areas (530–700 Å^2^), with TBAΔT12 (PDB entry 4lz1) [82] and TBA-T4W (PDB entry 6eo6) [83] showing the smallest and the largest interacting surfaces, respectively. Although the number of complexes with the ligand bound to exosite II is too limited to draw general conclusions, also in this case, the largest surfaces are exhibited by thrombin partners simultaneously anchoring the exosite and the active site (Figure 7b and Table 5). Among ligands that exclusively bind the exosite II, the largest interacting interfaces (~1000 Å^2^) are exhibited by the aptamers Toggle-25t/AF113-18 (PDB entry 5do4) [88] and HD22_27mer (PDB entry 4i7y) [89] and by the protein platelet glycoprotein Ibα (PDB entry 1p8v) [118]. Remarkable surfaces (750–800 Å^2^) are also exhibited by the natural ligands fibrinogen γ’ peptide (PDB entry 2hwl) [120] and by the kringle-2 domain (PDB entries 2hpp and 2hpq) [43]. On the other hand, rather limited interfaces are presented by heparin (PDB entries 1xmn and 1tb6) [115,116] and suramin (PDB entry 2h9t) [74]. In line with these observations, the classification of the interface areas as function of the ligand type (functional partners, natural inhibitors, and synthetic compounds) indicates that in all cases a wide distribution of values is observed (Figure S4).

**Figure 8:**
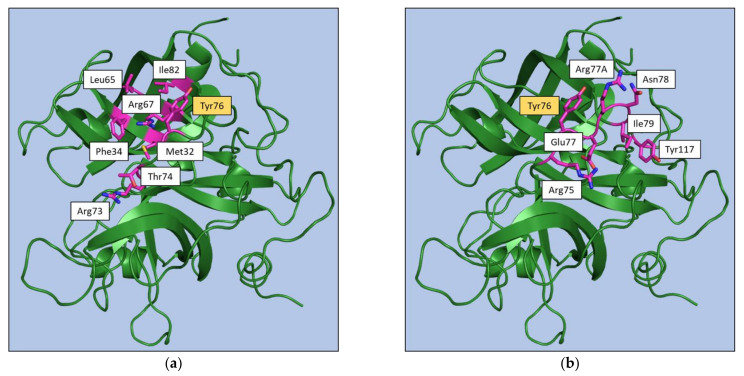
Exosite I residues involved in interaction of thrombin with at least the 50% of (**a**) non-aptamer or (**b**) aptamer ligands. Tyr76 is highlighted in yellow as it has been found in both classes.

To gain further insights into the anatomy of the exosite structure and into the residues that play key roles in ligand recognition, we monitored the residues that were buried upon complex formation and those that were involved in hydrogen bonding and electrostatic interactions (Tables S3 and S4). As shown in Table S5, Tyr76 is significantly buried (more than 70% of its surface) in the vast majority of the complexes formed by ligands binding to the exosite I (39 out of 46). The role of this residue is particularly evident in the complexes formed by the aptamers as Tyr76 is significantly buried in all the 9 complexes. Notably, the inspection of Table S5 highlights the different roles played by exosite I residues in anchoring aptamers and other ligands. In particular, Met32, Phe34, Leu65, Arg67, Thr74, and Ile82 are frequently involved in the binding of non-aptamer ligands while they play a marginal, if any, role in the recognition of aptamers (Figure 8). These latter ligands, in addition to Tyr76, preferentially bind to Arg75, Glu77, Arg77A, and Ile79. Residues of the region 73–77A also form hydrogen bonds and electrostatic interactions with both aptamer and non-aptamer ligands (Table S6). As specific features, Arg73 forms these interactions only with non-aptamers, whereas aptamers generally also bind Asn78 and Tyr117.

**Figure 9:**
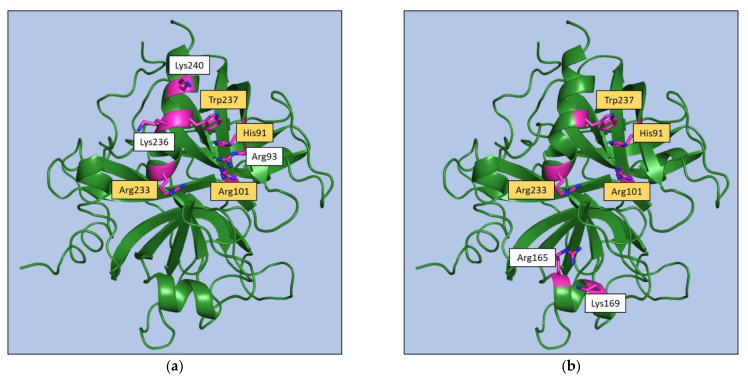
Exosite II residues involved in interaction of thrombin with (**a**) at least the 50% of the non-aptamer ligands or (**b**) the two aptamers bound to this exosite. Residues that have been found in both classes are highlighted in yellow.

Regarding the exosite II, the inspection of Table S7 indicates that key anchoring residues (His91, Arg93, and Trp237) may be identified for non-aptamer ligands, whereas non-conservative recognition patterns may be identified for aptamers that present distinctive bindings at this exosite (Figure 9). The same behavior emerges from the analysis of hydrogen bonding and electrostatic interactions (Table S8). Indeed, for non-aptamers, interactions with Arg93, Arg101, Arg233, and Lys236 are found in most of the complexes, whereas for aptamers the same residue rarely interacts with more than one aptamer.

Collectively, the survey of the available structural data for exosite-binding to thrombin clearly highlights some specificities of aptamer recognition within a conserved binding pattern (Figure 8 and Figure 9).

## 4. Beyond a Static View of Thrombin: Functional and Structural Evidence of Exosite Communication

In addition to the extensive experimental analyses of thrombin three-dimensional structure, significant contributions to the elucidation of structure-function relationships have been achieved through computational studies focused on the characterization of the protein dynamics.

Initial fully atomistic molecular dynamics (MD) simulations performed on the ligand-free form of thrombin in explicit water highlighted the dynamic behavior of the protein that was shown to be able to switch between an open state, likely related to the “fast form”, and a more compact conformation, possibly related to the “slow form” [130]. The remarkable dynamic propensity of thrombin and its correlation to the activity have been corroborated by later studies that have unraveled conformational states that were not present in the ensemble of the crystallographic structures [131]. In addition, these studies have underscored functional correlated motions between the active site and distant protein regions [132]. MD simulations have also provided interesting insights into the effect produced by the binding of monovalent cations, such as Li^+^, Na^+^, and Cs^+^, on the protein structure/function [133,134,135,136,137,138,139,140]. Moreover, several MD studies have been focused on the role of the flexibility on the substrate/inhibitor binding at the active site [141,142,143], on the structural impact of thrombin mutations [140,144], and on the structural basis of the aptamer recognition [145,146,147].

Experimental reports of the thrombin exosite long-range communications emerged from the analysis of the binding affinities of ligands belonging to different chemical species and from the variation of the biophysical properties of one exosite after a ligand binding at the other. Indeed, evidence of the occurrence of long-range mutual effects between the two exosites has been obtained by using a repertoire of different biophysical techniques (fluorescence, hydrogen-deuterium exchange/NMR, SPR, backscattering interferometry, and FIRMS) [106,107,108,110,148,149,150,151,152,153,154]. A qualitative summary of the outcome of these experiments is reported in Table S9. The binding of hirudin at the exosite I reduces the affinity at the exosite II for different ligands (γʹ-peptide and sF2, a synthetic peptide corresponding to residues 63–116 of prothrombin fragment 2) [149,153]. Equivalently, the binding of sF2 at exosite II reduces the affinity of hirudin at exosite I [149]. The binding affinity of the γʹ-peptide at the exosite II is also reduced upon the binding of the TBA aptamer at exosite I [153]. Notably, the effect of the TBA is dependent on the ligand at the exosite II. Indeed, the binding of the TBA aptamer at the exosite I increases the affinity of the HD22 aptamers at the exosite II [108,110]. As for the hirudin/sF2 pair of ligands [149], the mutual interplay between the exosites operates in both directions also for the TBA/HD22 ligands [108,110]. For other exosite II ligands, the effects produced on the affinity at the exosite I are varied. While the triply phosphorylated GpIbα (269−282, 3Yp) increases the affinity of both PAR1 and PAR3 [107], the binding of fibrin and TM456, a thrombomodulin-derived peptide, at exosite I is reduced by the association of different ligands at the exosite II [106,153].

It has been shown that this cross-talk between the two exosites may have important consequences on the inhibitory activity of some thrombin ligands. Indeed, it has been reported by different research groups that the binding of an aptamer at the exosite II may increase the anticoagulant activity of aptamers targeting the exosite I [112,155,156,157].

**Figure 10:**
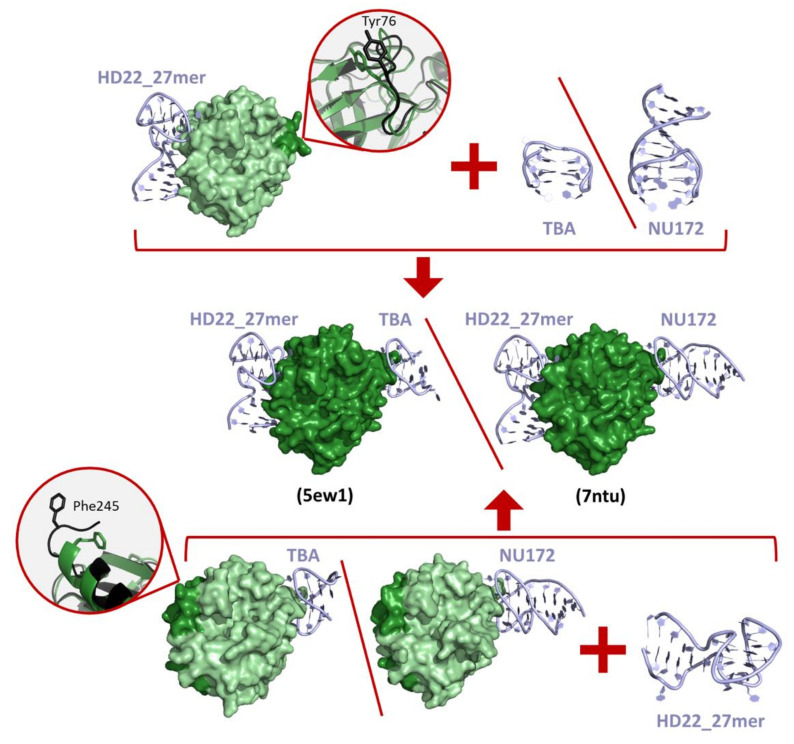
Simultaneous binding of TBA or NU172 (exosite I) and HD22_27mer (exosite II) to thrombin. MD studies [111,112] have shown that the HD22_27mer binding at the exosite II favors conformations of exosite I that are prone to the TBA/NU172 association (on the top) and vice versa (on the bottom). The conformational variations of the exosite I region between free (black) and HD22_27mer-bound (dark green) thrombin is highlighted in the inset on the top. Similarly, the conformational rearrangements of the protein C-terminus belonging to the exosite II between free (black) and TBA-bound or NU172-bound (dark green) thrombin is highlighted in the inset on the bottom. The crystallographic structures of the ternary complexes are shown in the middle (the PDB entries are reported within brackets).

Despite the remarkable accumulation of experimental data on the cross-talk between thrombin exosites, its structural characterization is conflicting [109,112]. In particular, small but significant conformational variations have been found at the exosite II-HD22_27mer interface only for the ternary complexes in which a TBA variants is bound to exosite I [109]. Long range inter-exosites communication can be related to the dynamic transmissions of the structural information from one exosite to the other. This hypothesis, also supported by hydrogen-deuterium exchange/NMR [150,151,152,154], has been corroborated by MD studies on thrombin-aptamer complexes carried out in recent years. Indeed, Xiao and Salsbury [158] found that the binding of the TBA aptamer to the exosite I has a significant impact on the conformational ensemble of thrombin by restricting the conformational freedom of the protein. On this basis, the authors suggested that conformational selection, i.e., generalized allostery, is the dominant mechanism of thrombin-aptamer binding. These findings have been corroborated and expanded on through extensive MD simulations of thrombin in different association states: ligand-free and binary/ternary complexes with the aptamers TBA (exosite I) and HD22_27mer (exosite II) [111]. These analyses clearly indicate that the HD22_27mer binding at the exosite II favors conformations of exosite I that are prone to TBA association (Figure 10). Similar effects are observed on the exosite II, which becomes conformationally prone to anchor HD22_27mer upon the binding of TBA to the exosite I (Figure 10). These results have been generalized by showing that a similar mechanism operates when the NU172 aptamer is bound to the exosite I [112] (Figure 10). Indeed, computational and experimental characterizations of the simultaneous binding of NU172 and HD22_27mer to thrombin have provided an atomic-level view of the synergistic action played by these two aptamers in promoting anticoagulant effects.

In conclusion, the MD studies carried out on thrombin-aptamer complexes perfectly fit into the conceptual framework, denoted as dynamic allostery [159], which assumes that the allosteric regulation does not produce discrete conformational rearrangements at the binding site but simply affects its dynamics. The previously unsuspected diffusion of this regulation mechanism has been recently underlined by Srinivasan and coworkers [160].

## 5. Conclusions

Although the first crystallographic characterizations of thrombin were reported more than thirty years ago, structural studies on this protein are continuously being performed. Indeed, the crucial role played by thrombin in the coagulation cascade and its multiple activities make it an attractive target for both basic and applied studies aimed at unravelling the basis of its many partnerships and at discovering new modulators with a therapeutic potential. Obviously, over the years, the focus of thrombin structural biology has been frequently shifted to cover different aspects of its many functions. In the last decade, the characterization of thrombin interactions with active site modulators has been associated with an extensive analysis of its interactions with aptamers, a class of compounds with a promising potential for both therapy and diagnosis of a wide range of human diseases [161]. Exploiting literature reports and the structural content of the PDB, we here surveyed the structural basis of thrombin-aptamer recognition in the framework of the binding modes that this protein exhibits in the recognition of functional, natural, and synthetic exosite binders. The present analysis clearly indicates that they tend to employ similar anchoring schemes, mainly dictated by their negatively charged groups, to the thrombin exosites. However, the binding mechanism of the aptamers is strictly related to the specific features of each exosite. A tight pincer-like bite determines the aptamer recognition of exosite I. On the contrary, the large surface of exosite II does not require a specific structural motif of the interacting aptamer as shown by the highly different structural organization of HD22_27mer and Toggle-25t. This overall structural view of thrombin partnerships here reported may be important for a comprehensive understanding of the role of the protein in the intricate blood coagulation process. It also provides insights for the design and development of novel inhibitors.

## Figures and Tables

**Table 1:** Functional interactors involved in the procoagulant and anticoagulant functions of thrombin.

Cofactor	Interactor/Substrate	Function
Heparin-like glycosaminoglycans (GAGs)	Antithrombin III	Thrombin inhibition
Heparin-like glycosaminoglycans (GAGs)	Heparin Cofactor II	Thrombin inhibition
-	Fibrinogen	Conversion to fibrin
Fibrin	Factor XIII	Activation to factor XIIIa, critical for stabilization of the clot through covalent cross-linking of the fibrin polymers
Thrombomodulin	Thrombin activatable fibrinolysis inhibitor (TAFI)	Activation of TAFI
Thrombomodulin	Protein C (PC)	Activation to activated protein C (APC) that inactivates cofactors Va and VIIIa
Platelet glycoprotein (GP) Ibα (GpIbα)	PAR-1	Cleavage of the platelet receptor PAR-1, resulting in platelet activation
Platelet glycoprotein (GP) Ibα (GpIbα)	Factor XI	Activation to factor XIa, stimulating its own generation
-	Factor V	Activation to factor Va, stimulating its own generation
-	Factor VIII	Activation to factor VIIIa, stimulating its own generation

**Table 2:** Natural (yellow) and synthetic (light blue) inhibitors of thrombin whose complexes have been structurally characterized.

Ligand	Description	Reference
AGAP008004-PA	Recombinant P5 fragment of cE5, a salivary protein from the major African malaria vector *Anopheles gambiae*	[55]
Anophelin	Anticoagulant from the malaria vector *Anopheles albimanus*	[56]
Avathrin	Thrombin inhibitor derived from a multicopy precursor in the salivary glands of the ixodid tick *Amblyomma variegatum*	[57]
Bivalirudin	Artificial anticoagulant peptide, mimic of hirudin, composed of 20 residues	[58]
Boophilin	Multifunctional kunitz-type proteinase inhibitor from the cattle tick *Rhipicephalus microplus*	[59]
CVS995 synthetic peptide	Divalent thrombin inhibitor that contains an alpha-keto-amide transition-state mimetic linking an active site binding group and a group that binds to the fibrinogen-binding exosite	[60]
Hemadin	Thrombin inhibitor from the land-living leech *Haemadipsa sylvestris*	[61]
Hirudin	Protein isolated from the salivary glands of the medicinal leech *Hirudo medicinalis*	[62]
Hirugen	N-acetyl-Hir53′-64′ with sulfato-Tyr63′, where Hir53′-64′ corresponds to the C-terminal residues of hirudin	[63]
Hirullin	A natural COOH-terminal peptide isolated from the leech *Hirudinaria manillensis*	[64]
Hirulog	Synthetic hirudin peptide analog	[63]
Hirunorm	Synthetic hirudin peptide analog	[65]
Hirutonin	Synthetic hirudin peptide analog	[66]
IgA fab	Randomly acquired antithrombin antibody found in a patient who presented a traumatic subdural hematoma	[67]
Madanin-1	Small cysteine-free thrombin inhibitor that facilitate blood feeding in the tick *Haemaphysalis longicornis*	[68]
Nonapeptide inhibitor	Synthetic hirudin peptide analog	[69]
Ornithodorin	Protein isolated from the blood sucking soft tick *Ornithodoros moubata*	[70]
Peptide inhibitor	C-terminal hirugen-like segment of a bivalent thrombin inhibitor	[71]
Rhodniin	Protein isolated from the assassin bug *Rhodnius prolixus*	[72]
Staphylocoagulase	Cofactor secreted by *Staphylococcus aureus* that activates prothrombin	[73]
Suramin	Hexasulfonated naphthylurea first made by O. Dressel, R. Kothe and B. Heymann at Bayer AG laboratories in Elberfeld	[74]
Synthetic inhibitor	Hirudin-based fibrinogen recognition exosite peptide inhibitor	[64]
Synthetic peptide	Synthetic peptide; sequence: Sin YEPI Hyp EE Smf Alc Q (Sin, succinic acid; Hyp, 4-hydroxyproline; Smf, 4-sulfomethyl-l-phenylalanine; and Alc, 2-amino-3-cyclohexyl-propionic acid)	[75]
Synthetic thrombin inhibitor P798	Synthetic bivalent thrombin inhibitor that comprises an active site blocking segment, a fibrinogen recognition exosite blocking segment, and a linker	[76]
Synthetic thrombin inhibitor P596	Bivalent peptidyl pyridinium methyl ketone inhibitor	[77]
Triabin	Protein from the saliva of the blood-sucking triatomine bug *Triatoma pallidipennis*	[30]
Tsetse thrombin inhibitor	Anticoagulant peptide produced in the salivary glands of the tsetse fly *Glossina morsitans*	[78]
Variegin	Thrombin inhibitor isolated from the tropical bont tick *Amblyomma variegatum*	[79]

**Table 3:** Aptamers targeting thrombin whose complexes have been structurally characterized.

Name	DNA or RNA	Sequence (5′ → 3′)	Reference
TBA	DNA	GGTTGGTGTGGTTGG	[80]
mTBA	DNA	3′-GGT-5′-5′-TGGTGTGGTTGG-3′	[81]
TBAΔT3	DNA	GG**N^Δ^**TGGTGTGGTTGG	[82]
TBAΔT12	DNA	GGTTGGTGTGG**N^Δ^**TGG	[82]
TBA-T4W	DNA	GGT**T^W^**GGTGTGGTTGG	[83]
TBA-T4K	DNA	GGT**T^K^**GGTGTGGTTGG	[83]
TBA-3L	DNA	GG**T^L^**TGGTGTGGTTGG	[84]
TBA-3G	DNA	GG**T^G^**TGGTGTGGTTGG	[84]
TBA-3Leu	DNA	GG**T^Leu^**TGGTGTGGTTGG	[84]
RE31	DNA	GTGACGTAGGTTGGTGTGGTTGGGGCGTCAC	[85]
NU172	DNA	CGCCTAGGTTGGGTAGGGTGGTGGCG	[86]
Toggle-25t	RNA	GGGAA**C^F^**AAAG**C^F^U^F^**GAAG**U^F^**A**C^F^U^F^U^F^**A**C^F^C^F^C^F^**	[87]
Toggle-25t/AF113-18	RNA	GGGAA**C^F^**AAAG**C^F^U^Se^**GAAG**U^FS2^**AC**U^F^U^Se^**A**C^F^C^F^C^F^**	[88]
HD22_27mer	DNA	GTCCGTGGTAGGGCAGGTTGGGGTGAC	[89]

**N^Δ^** represents abasic nucleotides; **T^W^** and **T^K^** represent C5-modified nucleotides; **T^L^**, **T^G^** and **T^Leu^** represent N3-modified nucleotides; **C^F^** and **U^F^** represent 2′-fluoropyrimidine nucleotides; **U^Se^** represents 2′-methylselenyluridine nucleotides; and **^S2^** represents phosphorodithioate linkage.

**Table 4:** Interaction data (interface area, number of H-bonds/salt bridges) detected in the crystallographic structures of thrombin complexes with ligands bound at exosite I or at both exosite I and active site. Functional partners, natural inhibitors, and synthetic compounds are highlighted in pink, yellow, and light blue, respectively. Analyses have been conducted using the PISA program [128].

Ligand (PDB Entry)	Interface Area (Å^2^)	N_HB_	N_SB_
**Exosite I**			
Coagulation Factor V (3p6z)	415.0	5	4
Human Factor V, A2-B domain linker (3p70)	297.4	2	0
Fibrinogen (2a45)	642.5	9	2
Platelet glycoprotein Ibα-GPIbα (1ook)	1060.0	17	10
Proteinase-activated receptor 1-PAR1 (1nrr)	401.1	4	0
Proteinase-activated receptor 3-PAR3 (2pux)	659.5	7	4
Thrombomodulin (1dx5)	772.7	12	0
Avathrin, C-terminal peptide (5gim)	516.6	3	1
IgA fab heavy chain (5e8e)	526.8	5	2
Hirugen (1aht)	627.1	7	3
Hirullin (1thr)	727.9	10	5
Staphylocoagulase (1nu7)	1661.9	22	15
Triabin (1avg)	697.4	7	5
mTBA (3qlp)	656.6	11	0
TBA (4dih)	565.0	12	0
TBAΔT12 (4lz1) *	533.8	13	0
TBA-T4W (6eo6) *	707.6	14	0
TBA-T4K (6eo7) *	640.6	13	0
TBA-3L (6z8v) *	605.8	14	0
TBA-3Leu (6z8x) *	592.2	9	0
RE31 (5cmx)	551.7	10	0
NU172 (6evv)	589.8	10	0
Bivalirudin, C-terminal fragment (3vxe)	566.5	6	1
Nonapeptide inhibitor (1g37)	512.0	2	0
Peptide inhibitor (1eb1)	544.8	4	3
Synthetic inhibitor (1ths) *	534.8	6	0
Synthetic peptide (2a2x) *	698.1	9	0
**Exosite I + active site**			
Heparin cofactor II (1jmo)	1743.8	30	13
Proteinase-activated receptor 1-PAR1 (3lu9)	1573.5	25	6
AGAP008004-PA from *A. gambiae* (5nhu)	1468.6	24	8
Anophelin (4e05)	1425.4	29	11
Boophilin (2ody)	1979.4	24	15
Hirudin variant 1 (2pw8)	1531.1	21	6
Hirudin variant 2 (4htc)	1652.1	20	15
Ornithodorin (1toc)	1763.3	15	7
Rhodniin (1tbr)	1703.7	16	10
Variegin (3b23)	900.0	12	5
CVS995 synthetic peptide (1dit)	1159.2	18	4
Hirulog (1abi)	1241.6	19	4
bza-2 hirulog (1qur) *	1348.3	13	2
Hirunorm IV (4thn)	1038.4	11	11
Hirunorm V (5gds)	1030.7	9	5
Hirutonin 2 (1ihs) *	1334.8	16	4
Hirutonin 6 (1iht) *	1027.2	14	4
Synthetic thrombin inhibitor P798 (1eoj) *	1188.8	8	4
Synthetic thrombin inhibitor P596 (1hbt)	1143.5	16	0

* PDB entries that were manually curated for the analyses.

**Table 5:** Interaction data (interface area, number of H-bonds/salt bridges) detected in the crystallographic structures of thrombin complexes with ligands bound at exosite II or at both exosite II and active site. Functional partners, natural inhibitors, and synthetic compounds are highlighted in pink, yellow, and light blue, respectively. Analyses have been conducted using the PISA program [128].

Ligand (PDB Entry)	Interface Area (Å^2^)	N_HB_	N_SB_
**Exosite II**			
Fibrinogen γ’ peptide (2hwl)	782.3	15	6
Bovine kringle-2 (2hpp)	795.2	15	9
Human kringle-2 (2hpq)	778.1	11	10
Heparin (1xmn) *	396.5	2	5
Heparin (in the presence of antithrombin III) (1tb6) *	307.9	0	6
Platelet glycoprotein Ibα-GPIbα (1p8v)	934.0	11	5
Toggle-25t/AF113-18 (5do4) *	924.9	17	0
HD22_27mer (4i7y)	1079.5	18	0
Suramin (2h9t)	476.0	3	0
**Exosite II + active site**			
Hemadin (1e0f)	1216.0	19	7
Madanin-1 (5l6n)	1365.3	24	13
Tsetse thrombin inhibitor (6tkl)	1652.4	27	11

* PDB entries that were manually curated for the analyses.

## Supplementary Materials

The following are available online at https://www.mdpi.com/article/10.3390/ijms221910803/s1.
