# Supplementary material for: Exosite Binding in Thrombin: A Global Structural/Dynamic Overview of Complexes with Aptamers and Other Ligands
Source: Int J Mol Sci. 2021 Oct 6;22(19):10803. doi: 10.3390/ijms221910803 (PMC8509272; doi:10.3390/ijms221910803)
Supplement: Supplementary file 1 [file ijms-22-10803-s001.zip › ijms-1392377-supplementary.pdf]

Supplementary material for:

Exosite binding in thrombin: a global structural/dynamic overview of complexes with aptamers and other ligands

Romualdo Troisi [1,†], Nicole Balasco [2,†], Ida Autiero [2,3], Luigi Vitagliano [2] and Filomena Sica [1,*]

[1] Department of Chemical Sciences, Complesso Universitario di Monte Sant'Angelo, University of Naples Federico II, Via Cintia, I-80126 Naples, Italy; romualdo.troisi@unina.it
[2] Institute of Biostructures and Bioimaging, CNR, I-80134 Naples, Italy; nicole.balasco@unina.it (N.B.); ida.autiero@gmail.com (I.A.); luigi.vitagliano@unina.it (L.V.)
[3] Molecular Horizon Srl, I-06084 Bettona, Italy
* Correspondence: filomena.sica@unina.it; Tel.: +39-081-674479
† These authors contributed equally to this work.

Table of contents

Figure S1. Cartoon representation of the crystallographic structure of TBA bound to the exosite I of thrombin – **page 2**
Figure S2. Cartoon representation of the crystallographic structure of HD22_27mer bound to the exosite II of thrombin – **page 3**
Figure S3. Cartoon representation of the crystallographic structure of NU172 bound to the exosite I of thrombin – **page 4**
Figure S4. Distribution of the interface areas found in the thrombin/ligand complexes as function of the ligand nature – **page 5**
Table S1. Amino acid residues representing the active site and the two exosites in the thrombin figures of this review – **page 6**
Table S2. List of the PDB entries containing at least one α-thrombin chain – **pages 7-8**
Table S3. Thrombin residues buried at different percentages at exosite I in the crystallographic structures of thrombin complexes – **pages 9-24**
Table S4. Thrombin residues buried at different percentages at exosite II in the crystallographic structures of thrombin complexes – **pages 25-28**
Table S5. Count of thrombin residues whose surface is buried at a percentage ≥70% upon complex formation with ligands bound at exosite I – **page 29**
Table S6. Count of thrombin residues involved in intermolecular interactions upon complex formation with ligands bound at exosite I – **page 30**
Table S7. Count of thrombin residues whose surface is buried at a percentage ≥70% upon complex formation with ligands bound at exosite II – **page 31**
Table S8. Count of thrombin residues involved in intermolecular interactions upon complex formation with ligands bound at exosite II – **page 32**
Table S9. Effect on the affinity of a ligand towards a thrombin exosite when another ligand is bound to the other exosite – **page 33**
References – page 34

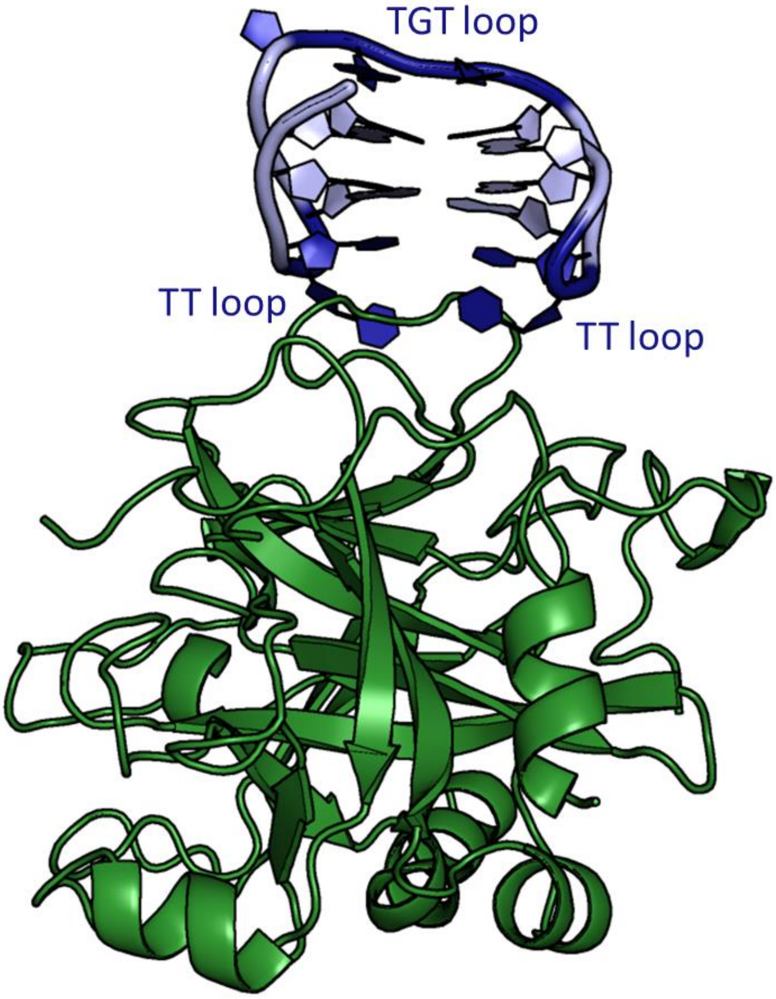

Figure S1. Cartoon representation of the crystallographic structure (PDB entry 4dih) of TBA (blue) bound to the exosite I of thrombin (green). The G-quadruplex loops of the aptamer are highlighted.

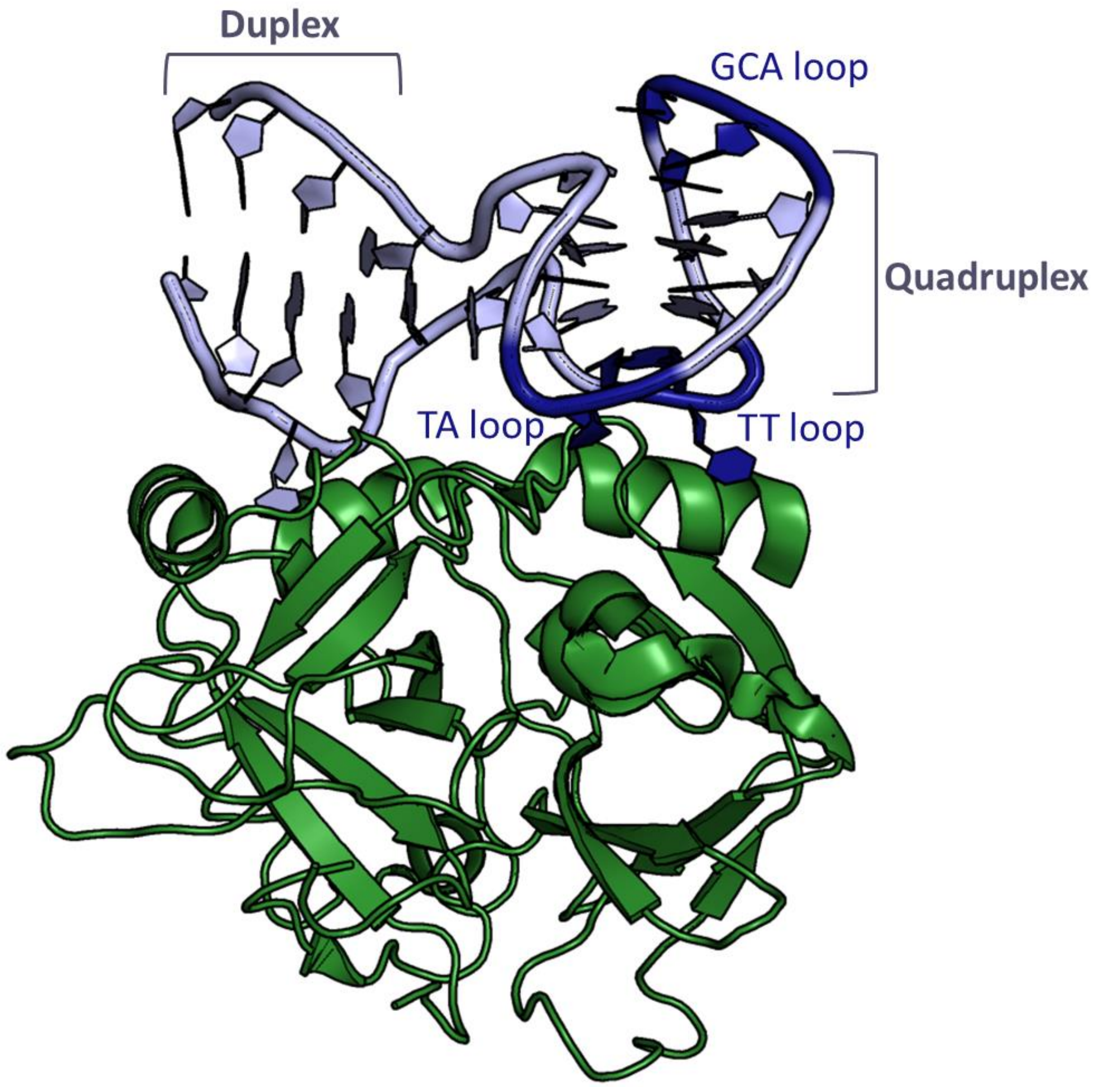

Figure S2. Cartoon representation of the crystallographic structure (PDB entry 4i7y) of HD22_27mer (blue) bound to the exosite II of thrombin (green). The loops of the G-quadruplex domain of the aptamer are highlighted.

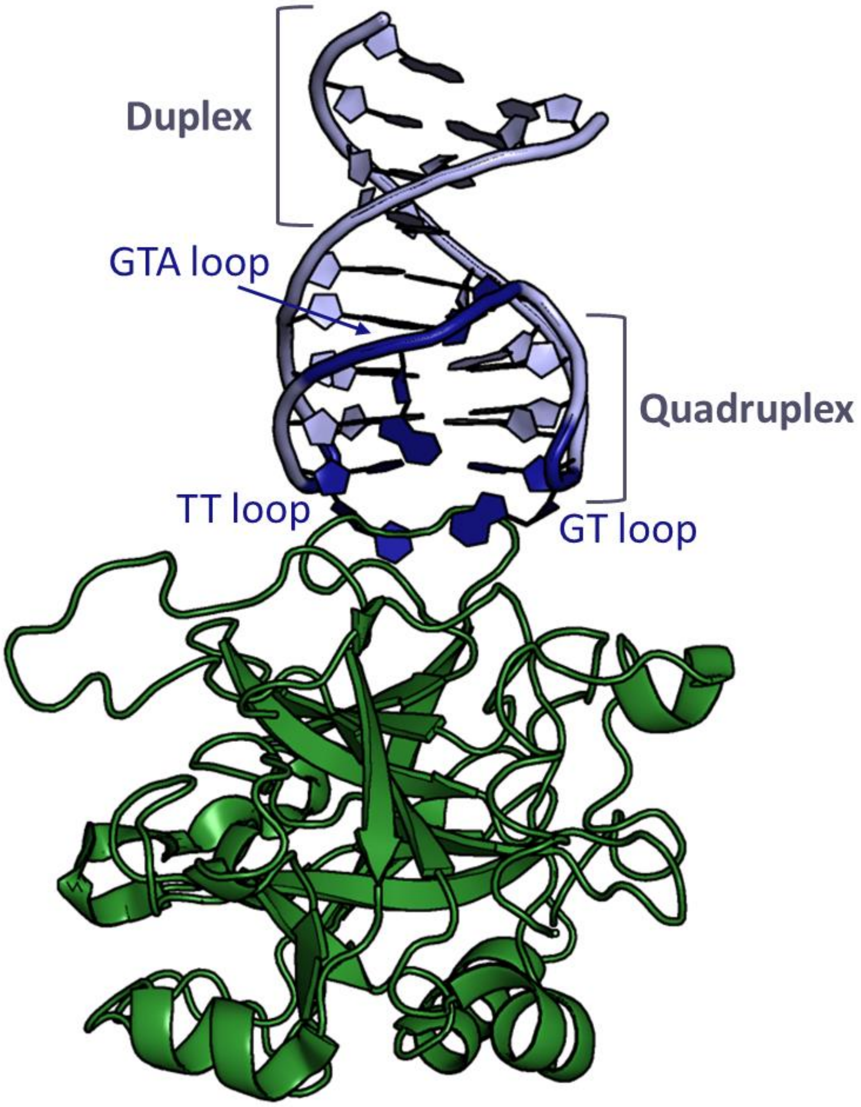

Figure S3. Cartoon representation of the crystallographic structure (PDB entry 6evv) of NU172 (blue) bound to the exosite I of thrombin (green). The loops of the G-quadruplex domain of the aptamer are highlighted.

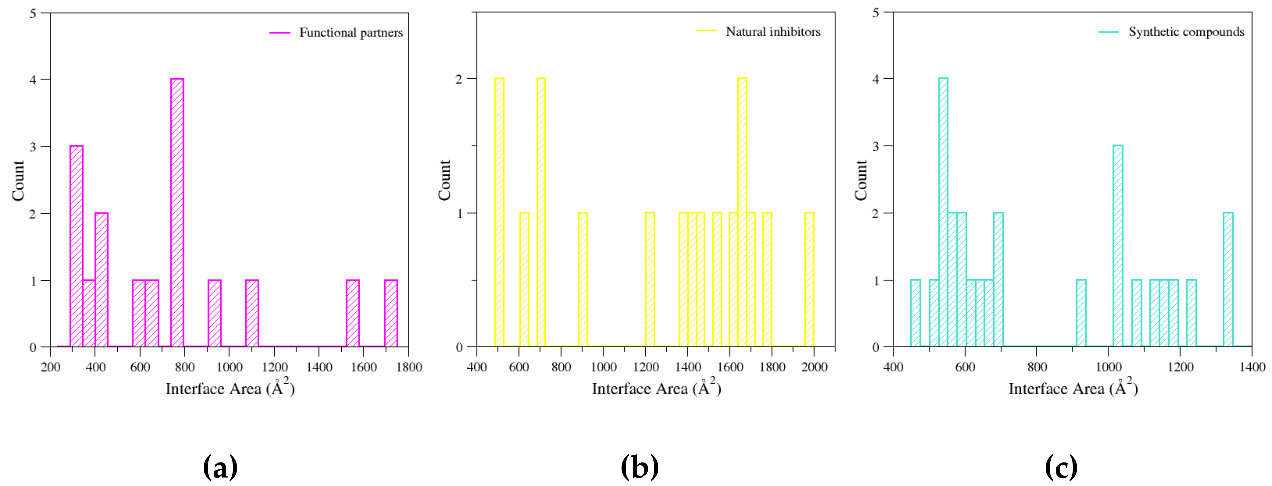

Figure S4. Distribution of the interface areas found in the thrombin/ligand complexes as function of the ligand nature: functional partners (a), natural inhibitors (b), and synthetic compounds (c). The criteria used to select the structures from the PDB are reported in the legend of Figure 7.

Table S1. Amino acid residues representing the active site and the two exosites in the thrombin figures of this review.

**Active site**	His57, Asp102, Ser195
**Exosite I**	Ile24, Arg73, Thr74, Arg75, Tyr76, Glu77, Arg77A, Asn78, Ile79, Ile82
**Exosite II**	Tyr89, Ile90, His91, Pro92, Arg93, Tyr94, Asn95, Trp96, Arg97, Glu97A, Asn98, Leu99, Asp100, Arg101, Arg126, Glu127, Thr128, Ala129, Ala129A, Ser129B, Leu129C, Leu130, Glu164, Arg165, Pro166, Val167, Cys168, Lys169, Phe232, Arg233, Leu234, Lys235, Lys236, Trp237, Ile238, Gln239, Lys240, Val241, Ile242, Asp243, Gln244, Phe245

Table S2. List of the PDB entries containing at least one α-thrombin chain (see legend of Figure 4).

Deposition year	Number of PDB entries	PDB entries
1991	3	1hgt, 1ppb, 2hgt
1992	10	1abi, 1abj, 1bbr, 1dwb, 1dwc, 1dwd, 1dwe, 1etr, 1ets, 1ett
1993	13	1fph, 1hrt, 1hut, 1ihs, 1iht, 1thr, 1ths, 1tmb, 2hpp, 2hpq, 3htc, 4h6t, 4htc
1994	20	1fpc, 1hag, 1hah, 1hai, 1hdt, 1hlt, 1lhc, 1lhd, 1lhe, 1lhf, 1lhg, 1nrn, 1nro, 1nrp, 1nrq, 1nrr, 1nrs, 1tmt, 1tmu, 3hat
1995	10	1aht, 1bmm, 1bmn, 1dit, 1hao, 1hap, 1hbt, 1hxe, 1tbq, 1tbr
1996	10	1bth, 1hxf, 1toc, 1tom, 1ucy, 1uma, 1uvs, 1uvt, 1uvu, 1vit
1997	15	1a0h, 1a2c, 1ad8, 1ae8, 1afe, 1ai8, 1aix, 1avg, 1awf, 1awh, 1ay6, 1mkw, 1mkx, 1ycp, 5gds
1998	14	1a3b, 1a3e, 1a46, 1a4w, 1a5g, 1a61, 1b5g, 1ba8, 1bb0, 1bcu, 1ca8, 1tbz, 1vr1, 4thn
1999	31	1b7x, 1c1u, 1c1v, 1c1w, 1c4u, 1c4v, 1c4y, 1c5l, 1c5n, 1c5o, 1d3d, 1d3p, 1d3q, 1d3t, 1d4p, 1d6w, 1d9i, 1de7, 1dm4, 1doj, 1dx5, 1qbv, 1qhr, 1qj1, 1qj6, 1qj7, 1qur, 1thp, 2thf, 7kme, 8kme
2000	6	1e0f, 1eoj, 1eol, 1g30, 1g32, 1g37
2001	15	1eb1, 1ghv, 1ghw, 1ghx, 1ghy, 1gj4, 1gj5, 1h8d, 1h8i, 1id5, 1jmo, 1jou, 1jwt, 1k21, 1k22
2002	6	1kts, 1ktt, 1mh0, 1mu6, 1mu8, 1mue
2003	14	1nm6, 1no9, 1nt1, 1nu7, 1nu9, 1ny2, 1nzq, 1o0d, 1o2g, 1o5g, 1ook, 1oyt, 1p8v, 1rd3
2004	21	1sb1, 1sfq, 1sg8, 1sgi, 1shh, 1sl3, 1sr5, 1t4u, 1t4v, 1ta2, 1ta6, 1tb6, 1tq0, 1tq7, 1twx, 1vzq, 1w7g, 1way, 1wbg, 1xm1, 1xmn
2005	34	1ype, 1ypg, 1ypj, 1ypk, 1ypl, 1ypm, 1z71, 1z8i, 1z8j, 1zgi, 1zgv, 1zrb, 2a0q, 2a1d, 2a2x, 2a45, 2afq, 2ank, 2anm, 2b5t, 2bdy, 2bvr, 2bvs, 2bvx, 2bxt, 2bxu, 2c8w, 2c8x, 2c8y, 2c8z, 2c90, 2c93, 2feq, 2fes
2006	10	2cf8, 2cf9, 2cn0, 2gde, 2gp9, 2h9t, 2hwl, 2ocv, 2od3, 2ody

2007	24	2jh0, 2jh5, 2jh6, 2pgb, 2pgq, 2pux, 2pv9, 2pw8, 2r2m, 2uuf, 2uuj, 2uuk, 2v3h, 2v3o, 2zc9, 2zda, 2zdv, 2zf0, 3b9f, 3bef, 3bei, 3bf6, 3biu, 3biv
2008	30	2zff, 2zfp, 2zfq, 2zfr, 2zg0, 2zgb, 2zgx, 2zhe, 2zhf, 2zhq, 2zhw, 2zi2, 2ziq, 2znk, 2zo3, 3bv9, 3c1k, 3c27, 3d49, 3da9, 3dd2, 3dhk, 3dt0, 3dux, 3e6p, 3edx, 3ee0, 3egk, 3eq0, 3f68
2009	9	3gic, 3gis, 3hk3, 3hk6, 3hki, 3hkj, 3jz1, 3jz2, 3k65
2010	8	3ldx, 3lu9, 3p17, 3p6z, 3p70, 3pma, 3pmb, 3pmh
2011	36	3b23, 3qdz, 3qgn, 3qlp, 3qto, 3qtv, 3qwc, 3qx5, 3r3g, 3rlw, 3rly, 3rm0, 3rm2, 3rml, 3rmm, 3rmn, 3rmo, 3s7h, 3s7k, 3sha, 3shc, 3si3, 3si4, 3sqe, 3sqh, 3sv2, 3t5f, 3tu7, 3u69, 3u8o, 3u8r, 3u8t, 3u98, 3u9a, 3utu, 3uwj
2012	22	3vxe, 3vxf, 4ax9, 4ayv, 4ayy, 4az2, 4bah, 4bak, 4bam, 4ban, 4bao, 4baq, 4dih, 4dii, 4dt7, 4dy7, 4e05, 4e06, 4e7r, 4h6s, 4hfp, 4i7y
2013	8	4boh, 4ch2, 4ch8, 4loy, 4lxb, 4lz1, 4lz4, 4mlf
2014	7	4rkj, 4rko, 4rn6, 4ud9, 4udw, 4ue7, 4ueh
2015	15	4ufd, 4ufe, 4uff, 4ufg, 4yes, 5a2m, 5af9, 5afy, 5afz, 5ahg, 5cmx, 5do4, 5e8e, 5ew1, 5ew2
2016	10	5gim, 5jdu, 5jfd, 5jzy, 5l6n, 5lce, 5lpd, 5mjt, 5mls, 5mm6
2017	6	5nhu, 6eo6, 6eo7, 6eo8, 6eo9, 6evv
2018	7	6cym, 6fjt, 6gbw, 6gn7, 6gwe, 6hsx, 6i51
2019	25	6p9u, 6px5, 6pxj, 6pxq, 6rot, 6t3m, 6t3q, 6t4a, 6t52, 6t53, 6t54, 6t55, 6t56, 6t57, 6t7h, 6t89, 6t8a, 6tdt, 6tkg, 6tkh, 6tki, 6tkj, 6tkl, 6v5t, 6v64
2020	12	6y02, 6z8v, 6z8w, 6z8x, 6zug, 6zuh, 6zun, 6zuu, 6zuw, 6zux, 6zv7, 6zv8

Table S3. Thrombin residues buried at different percentages (≥70%, 30-70%, 10-30%) upon complex formation with ligands bound at exosite I or at both exosite I and active site in the crystallographic structures of thrombin complexes. The protein residues involved in intermolecular hydrogen bonds (HB) and salt bridges (SB) are also reported. Functional partners, natural inhibitors, and synthetic compounds are highlighted in pink, yellow, and light blue, respectively. Analyses have been conducted using the PISA program [1].

Ligand (PDB entry)	≥ 70%	< 70% and > 30%	≤ 30% and ≥ 10%	HB	SB
**Exosite I**					
Coagulation Factor V (3p6z)	Met32, Arg73, Thr74, Tyr76	Phe34, Gln38, Arg67	Glu39, Glu40, Arg75, Arg77A, Ile82, Gln151	Gln38, Thr74, Arg75, Tyr76	Arg73, Arg75, Arg77A
Human Factor V, A2-B domain linker (3p70)	Met32, Thr74, Tyr76	Phe34, Arg67	Gln38, Glu39, Leu40, Arg73, Arg75, Arg77A, Ile82	Thr74, Tyr76	-
Fibrinogen (2a45)	Lys36, Leu65, Arg67, Tyr76, Arg77A, Ile82	Phe34, Ser36A, Glu38, Glu80, Met84	Arg35, Arg75, Asn78, Lys81, Ser83, Lys109, Lys110	Lys36, Ser36A, Glu38, Tyr76, Arg77A, Ile82, Met84	Lys110, Arg77A
Platelet glycoprotein Ib*α* - GPIb*α* (1ook)	Pro37, Gln38, Leu65, Tyr76, Met84, Thr147, Gly150, Gln151, Pro152, Ser153	Lys36, Ser36A, Glu39, Arg67, Arg77A, Lys81, Ile82, Lys145, Lys149E,	Phe34, Arg35, Trp60D, Asp60E, Asn62, Arg73, Thr74, Asn78, Lys110, Asn143, Leu144, Trp148,	Ser36A, Gln38, Glu39, Arg73, Arg77A, Lys81, Met84, Lys145, Thr147, Gly150, Gln151, Ser153	Arg73, Arg77A, Lys110, Lys145

			Gly149D		
Proteinase-activated receptor 1 - PAR1 (1nrr)	Met32, Phe34, Arg67, Arg73, Thr74	Gln38, Leu65, Tyr76	Glu39, Leu40, Arg75, Arg77A, Ile82, Gln151	Leu40, Arg73, Thr74	-
Proteinase-activated receptor 3 - PAR3 (2pux)	Met32, Phe34, Leu65, Arg67, Thr74, Tyr76, Ile82, Ser83	Gln38, Arg73, Lys81, Met84, Val112	Glu39, Leu40, Arg75, Arg77A, Lys110, Pro111, Pro113, Gln151	Thr74, Arg75, Tyr76, Arg77A, Ile82, Lys110	Arg75, Arg77A
Thrombomodulin (1dx5)	Pro37, Gln38, Arg67, Tyr76, Ile82	Phe34, Leu65, Thr74, Arg77A, Glu80, Lys81, Mey84, Lys110	Lys36, Ser36A, Glu39, Arg75, Asn78, Gln151	Ser36A, Gln38, Thr74, Arg75, Tyr76, Arg77A, Lys81, Ile82	-
Avathrin, C-terminal peptide (5gim)	Met32, Phe34, Leu65, Arg67, Thr74, Tyr76, Ile82	Gln38, Arg73	Glu39, Leu40, Arg75, Ser83, Met84, Gln151	Thr74, Arg75, Tyr76	Arg75
IgA fab heavy chain (5e8e)	Met32, Phe34, Gln38, Arg67, Arg73, Thr74, Tyr76	Leu40, Leu65, Arg77A, Ile82	Ser36A, Pro37, Glu39, Arg75, Gln151	Gln38, Thr74, Tyr76, Arg77A	Arg73
Hirugen (1aht)	Met32, Phe34, Leu65, Arg67, Arg73,	Lys36, Gln38, Arg75, Glu80, Met84	Ser36A, Glu39, Leu40, Lys81, Gln151	Arg73, Thr74, Arg75, Tyr76, Ile82	Arg73

	Thr74, Tyr76, Ile82				
Hirullin (1thr)	Met32, Phe34, Leu65, Arg67, Thr74, Tyr76, Ile82, Ser83	Gln38, Arg73, Lys81, Met84, Lys110, Val112	Lys36, Glu39, Leu40, Arg75, Lys109, Pro111, Ala113, Gln151	Arg73, Thr74, Tyr76, Ile82, Ser83, Lys110, Pro111	Arg73, Lys110
Staphylocoagulase (1nu7)	Gln38, Leu65, Arg67, Arg75, Tyr76, Arg77A, Ile82, Met84, Lys145, Glu146, Trp148, Val149C, Gly150, Arg221	Phe34, Arg73, Thr74, Glu80, Lys81, Ser83, Lys110, Leu144, Thr147, Thr149, Gln151, Ser153, Val154	Asp21, Lys36, Ser36A, Pro37, Asn78, Lys109, Asn149B, Gly149D, Lys149E, Arg173, Asp186A, Gly219, Cys220, Asp221A, Asp222	Gln38, Arg73, Thr74, Arg75, Arg77A, Lys81, Met84, Lys110, Lys145, Trp148	Asp21, Arg73, Arg75, Arg77A, Lys81, Lys110, Lys145
Triabin (1avg)	Lys36, Ser36A, Gln38, Leu65, Arg67, Tyr76, Arg77A, Met84	Phe34, Ile82	Pro37, Asp62, Thr74, Arg75, Lys78, Lys81, Lys109, Lys110	Lys36, Ser36A, Gln38, Thr74, Arg77A	Arg77A
mTBA (3qlp)	Leu65, Arg75, Tyr76, Glu77, Arg77A, Ile79	Ile24, Asn78, Ile82, Tyr117	Lys36, Gln38, Arg67, Gly69, His71, Ser72, Thr74	Arg75, Tyr76, Glu77, Arg77A, Asn78, Tyr117	-
TBA (4dih)	Arg75, Tyr76,	Ile24, Tyr117	Gln38, Gly69,	Arg75, Tyr76,	-

	Glu77,<br>Arg77A,<br>Ile79		His71,<br>Ser72,<br>Thr74,<br>Asn78,<br>Ile82	Glu77,<br>Arg77A,<br>Tyr117	
TBAΔT12 (4lz1)*	Arg75,<br>Tyr76,<br>Glu77,<br>Arg77A,<br>Ile79	Ile24,<br>Tyr117	Gln38,<br>Arg67,<br>Gly69,<br>His71,<br>Ser72,<br>Thr74,<br>Asn78,<br>Ile82	Arg75,<br>Tyr76,<br>Glu77,<br>Arg77A,<br>Asn78<br>Tyr117	-
TBA-T4W (6eo6)*	Leu65,<br>Arg67,<br>Arg75,<br>Tyr76,<br>Glu77,<br>Arg77A,<br>Ile79	Ile24,<br>Phe34,<br>Gln38,<br>His71,<br>Thr74,<br>Asn78,<br>Ile82,<br>Tyr117	Lys36,<br>Gly69,<br>Ser72,<br>Val154	Thr74,<br>Arg75,<br>Tyr76,<br>Glu77,<br>Arg77A,<br>Asn78,<br>Tyr117	-
TBA-T4K (6eo7)*	Arg75,<br>Tyr76,<br>Glu77,<br>Arg77A,<br>Ile79	Ile24,<br>Leu65,<br>His71,<br>Thr74,<br>Ile82,<br>Tyr117	Gln38,<br>Gly69,<br>Ser72,<br>Asn78,<br>Val154	Arg75,<br>Tyr76,<br>Glu77,<br>Arg77A,<br>Asn78,<br>Tyr117	-
TBA-3L (6z8v)*	Arg75,<br>Tyr76,<br>Glu77,<br>Arg77A,<br>Ile79	Ile24,<br>His71,<br>Ile82,<br>Tyr117	Gln38,<br>Leu65,<br>Arg67,<br>Gly69,<br>Ser72,<br>Thr74,<br>Asn78,<br>Met84,<br>Val154	Thr74,<br>Arg75,<br>Tyr76,<br>Glu77,<br>Arg77A,<br>Asn78,<br>Tyr117	-
TBA-3Leu (6z8x)*	Arg75,<br>Tyr76,<br>Glu77,<br>Arg77A,<br>Ile79	Ile24,<br>His71,<br>Ile82,<br>Tyr117	Ser72,<br>Thr74,<br>Asn78	His71,<br>Arg75,<br>Tyr76,<br>Arg77A,<br>Asn78,<br>Tyr117	-

RE31 (5cmx)	Arg75, Tyr76, Glu77, Arg77A, Ile79	Ile24, His71, Asn78, Tyr117	Gly69, Ser72, Thr74, Ile82, Val154	Thr74, Arg75, Tyr76, Glu77, Arg77A, Tyr117	-
NU172 (6evv)	Arg75, Tyr76, Glu77, Ile79	Ile24, His71, Ser72, Arg77A, Asn78, Ile82, Tyr117	Gly69, Thr74	Gly69, Arg75, Tyr76, Glu77, Arg77A	-
Bivalirudin, C-terminal fragment (3vxe)	Met32, Phe34, Leu65, Arg67, Thr74, Tyr76, Ile82	Lys36, Gln38, Arg73, Met84	Glu39, Leu40, Arg75, Arg77A	Gln38, Thr74, Arg75, Tyr76	Arg75
Nonapeptide inhibitor (1g37)	Met32, Phe34, Gln38, Leu65, Arg67, Tyr76, Ile82	Lys36, Thr74	Ser36A, Glu39, Leu40, Arg73, Arg75, Ser83, Met84	Thr74, Tyr76	-
Peptide inhibitor (1eb1)	Met32, Phe34, Leu65, Arg67, Arg73, Thr74, Tyr76, Ile82	Gln38, Leu40	Lys36, Glu39, Arg75, Met84, Asn149B, Gln151	Arg73, Thr74, Tyr76	Arg73
Synthetic inhibitor (1ths)*	Met32, Phe34, Leu65, Arg67, Arg73, Thr74, Tyr76, Ile82	Gln38, Leu40, Met84	Lys36, Glu39, Arg75, Arg77A, Lys110, Gln151	Arg73, Thr74	-

Synthetic peptide (2a2x)*	Met32, Phe34, Lys36, Arg67, Arg73, Thr74, Tyr76, Glu80, Ile82	Gln38, Leu40, Leu64, Leu65, Met84	Glu39, Glu61, Asn62, Arg75, Asn78, Lys81, Lys109, Gln151	Gln38, Asn62, Arg73, Thr74	-
**Exosite I + active site**					
Heparin cofactor II (1jmo)	Ser36A, Pro37, Gln38, Leu41, Cys42, His57, Tyr60A, Trp60D, Leu65, Arg67, Ser72, Thr74, Ile82, Asn98, Leu99, Trp147A, Ala190, Cys191, Glu192, Gly193, Ala195, Val213, Ser214, Trp215, Gly216, Glu217, Gly219, Cys220, Gly226	Asp60E, Lys60F, Arg75, Tyr76, Ser83, Met84, Lys110, Asn147D, Ser153, Ile174	Phe34, Lys36, Glu39, Leu40, Pro60C, Arg73, Arg77A, Lys81, Trp96, Glu97A, Asn143, Glu146, Ala147C, Val147E, Gly148, Lys149, Val154, Asp189, Arg221A, Phe227	Ser36A, Gln38, Leu40, Leu41, Pro60C, Lys60F, Ser72, Thr74, Arg75, Tyr76, Lys110, Trp147A, Asn147D, Glu192, Gly193, Ala195, Ser214, Gly216, Glu217, Arg221A	Asp60E, Arg75, Lys110, Glu192
Proteinase-activated receptor 1 - PAR1 (3lu9)	Met32, Phe34, Gln38, Glu39,	Tyr60A, Trp60D, Lys60F, Tyr76,	Arg35, Lys36, Pro37, Arg75,	Gln38, Glu39, Leu40, Leu41,	Arg75, Asp189

	Leu40, Leu41, Cys42, His57, Leu65, Arg67, Arg73, Thr74, Ile82, Asn143, Gln151, Ile174, Ala190, Cys191, Glu192, Gly193, Ala195, Val213, Ser214, Trp215, Gly216, Glu217, Gly219, Gly226	Met84, Glu97A, Asn98, Leu99, Arg173, Asp189, Tyr228	Ser83, Thr147, Lys149E, Gly150, Arg175, Cys220, Arg221A, Tyr225, Phe227	Arg73, Thr74, Arg75, Tyr76, Ile82, Lys149E, Gln151, Arg173, Asp189, Gly193, Ala195, Ser214, Gly216, Glu217, Gly219	
AGAP008004-PA from *A. gambiae* (5nhu)	Phe34, Gln38, Glu39, Leu40, Leu41, His57, Tyr60A, Leu65, Arg67, Ile82, Met84, Asn98, Leu99, Gly142, Ile174, Ala190, Cys191, Glu192, Gly193,	Cys42, Trp60D, Lys60F, Arg73, Thr74, Tyr76, Lys110, Trp141, Asn143, Gln151, Asp189, Ser214, Glu217, Cys220	Lys36, Pro37, Pro60C, Ser83, Trp96, Arg97, Glu97A, Lys109, Arg173, Tyr225, Phe227	Gln38, Leu40, His57, Tyr60A, Trp60D, Arg73, Met84, Gln151, Arg173, Asp189, Glu192, Gly193, Ser195, Gly216, Gly219	His57, Arg73, Asp189

	Ser195, Val213, Trp215, Gly216, Gly219, Gly226, Tyr228				
Anophelin (4e05)	Met32, Phe34, Glu39, Leu40, Leu41, His57, Tyr60A, Arg67, Arg73, Thr74, Asn98, Leu99, Gly142, Asn143, Ala190, Cys191, Glu192, Gly193, Ser195, Val213, Trp215, Gly216, Glu217, Gly219, Gly226, Tyr228	Gln38, Cys42, Trp60D, Lys60F, Tyr76, Ile82, Trp141, Gln151, Ile174, Asp189, Ser214, Cys220, Arg221, Lys224	Cys58, Leu65, Arg75, Arg77A, Met84, Trp96, Glu97A, Glu146, Thr147, Lys147G, Thr172, Arg173, Tyr225, Phe227	Gln38, Leu40, His57, Arg73, Tyr76, Glu146, Lys147G, Gln151, Asp189, Ala190, Glu192, Gly193, Ser195, Trp215, Gly216, Glu217, Gly219, Arg221, Lys224	His57, Glu146, Asp189, Glu192, Lys224
Boophilin (2ody)	Met32, Phe34, Gln38, Glu39, Leu41, Cys42, His57, Tyr60A, Pro60C, Trp60D,	Leu40, Lys60F, Glu80, Lys81, Ser83, Met84, Lys97, Arg110, Gln151, Cys220	Arg35, Lys36, Pro37, Cys58, Phe60H, Arg75, Lys78, Trp96, Glu97A, Glu113,	Glu39, Tyr60A, Trp60D, Arg73, Thr74, Tyr76, Arg77A, Lys81, Ile82, Met84,	Glu39, Arg73, Lys81, Arg110, Asp189, Glu192, Lys224

	Leu65, Arg67, Arg73, Thr74, Tyr76, Arg77A, Ile82, Asn98, Leu99, Trp148, Val149C, Ile174, Ala190, Cys191, Glu192, Ser195, Val213, Ser214, Trp215, Gly216, Glu217, Gly219, Gly226, Phe227, Tyr228		Asn143, Glu146, Thr147, Thr149, Ala149D, Arg173, Asp189, Gly193, Arg221A, Lys224	Arg110, Trp148, Asp189, Glu192, Ser195, Trp215, Gly216, Glu217, Gly219	
Hirudin variant 1 (2pw8)	Met32, Phe34, Lys36, Gln38, His57, Tyr60A, Pro60C, Trp60D, Leu65, Arg67, Arg73, Thr74, Tyr76, Ile82, Leu99, Glu192, Ser195, Trp215,	Leu40, Lys60F, Glu80, Glu146, Ile174, Cys191, Ser214, Cys220, Arg221A, Lys224	Glu39, Leu41, Asn62, Arg75, Arg77I, Asn78, Lys81, Met84, Trp96, Arg97, Glu97A, Asn98, Asn143, Thr147, Gln151, Thr172, Arg173	Trp60D, Lys60F, Arg73, Thr74, Tyr76, Lys81, Ile82, Trp96, Arg97, Arg173, Glu192, Ser195, Ser214 Gly216, Glu217, Gly219, Cys220, Arg221A	Arg73, Arg173, Arg221A

	Gly216, Glu217, Gly219				
Hirudin variant 2 (4htc)	Met32, Phe34, Lys36, Glu39, Leu40, His57, Tyr60A, Pro60C, Trp60D, Leu65, Arg67, Arg73, Thr74, Tyr76, Ile82, Leu99, Gly142, Asn143, Cys191, Glu192, Ser214, Trp215, Gly216, Glu217, Gly219	Gln38, Lys60F, Glu97A, Glu146, Gln151, Arg173, Ile174, Gly193, Ser195, Cys220, Arg221A, Lys224	Arg35, Pro37, Leu41, Arg75, Arg77A, Met84, Trp96, Arg97, Asn98, Trp141, Thr147, Trp148, Lys149E, Ser171, Thr172, Ala190	Lys36, Leu40, Trp60D, Lys60F, Arg73, Tyr76, Arg173, Glu192, Ser195, Ser214, Gly216, Glu217, Gly219, Arg221A, Lys224	Glu39, Lys60F, Arg73, Arg77A, Lys149E, Arg173, Arg221A
Ornithodorin (1toc)	Phe34, Gln38, Leu41, His57, Tyr60A, Trp60D, Leu65, Arg67, Thr74, Tyr76, Arg77A, Ile82, Leu99, Asn143, Glu146,	Lys36, Glu39, Leu40, Pro60C, Arg73, Met84, Trp148, Ile174, Arg221	Ser37, Asp60E, Lys60F, Arg75, Glu80, Lys81, Ser83, Arg145, Thr149A, Arg173, Asp189, Cys220, Lys224	Ser37, Tyr60A, Pro60C, Trp60D, Asp60E, Arg77A, Ile82, Glu146, Trp148, Cys191, Glu192, Ser214, Gly216, Gly219	Arg67, Arg73, Arg77A

	Thr147, Gln151, Ala190, Cys191, Glu192, Gly193, Ser195, Val213, Ser214, Trp215, Gly216, Glu217, Gly219				
Rhodniin (1tbr)	Glu39, Leu41, Cys42, His57, Cys58, Trp60D, Lys60F, Leu65, Arg67, Thr74, Tyr76, Gly142, Asn143, Trp148, Ala190, Cys191, Glu192, Gly193, Ser195, Val213, Ser214, Trp215, Gly216, Glu217	Phe34, Arg35, Lys36, Gln38, Leu40, Tyr60A, Arg75, Arg77A, Ile82, Met84, Leu99, Thr147, Ile174, Gly219, Gly226	Ser37, Pro37A, Tyr60A, Pro60C, Phe60H, Arg73, Glu77, Lys78, Lys109, Arg110, Glu146, Gln151, Arg173, Asp189, Cys220, Arg221, Lys224	Glu39, Leu41, Lys60F, Thr74, Tyr76, Arg77A, Trp148, Glu192, Gly193, Ser195	Glu39, Arg77A, Lys109, Arg110, Glu192, Ser214, Gly216, Gly219
Variegin (3b23)	Met32, Phe34, Leu40, Leu41, Cys42, Cys58,	Gln38, Glu39, His57, Leu65, Lys81, Ile82,	Arg35, Trp60D, Arg75, Arg77A, Met84, Lys110,	Gln38, Glu39, Thr74, Tyr76, Arg77A, Asn143,	Glu39, Arg77A, Glu192

	Lys60F, Arg67, Arg73, Thr74, Tyr76, Gly142, Asn143, Gly193	Trp141, Gln151, Glu192	Thr147, Ser195	Gln151, Glu192, Ser195	
CVS995 synthetic peptide (1dit)	Met32, Phe34, Gln38, Leu40, Cys42, His57, Leu65, Arg67, Arg73, Thr74, Asn98, Gly142, Ala190, Cys191, Gly193, Ser195, Val213, Trp215, Gly216, Gly219, Gly226	Pro37, Glu39, Leu41, Tyr60A, Lys60F, Tyr76, Leu99, Trp141, Gln151, Ile174, Asp189, Glu192, Ser214, Glu217, Tyr228	Lys36, Trp60D, Arg75, Ile82, Glu97A, Asn143, Cys220, Arg221A, Tyr225, Phe227	Leu40, His57, Lys60F, Arg73, Thr74, Gln151, Asp189, Gly193, Ser195, Ser214, Gly216, Gly219	Arg73
Hirulog (1abi)	Met32, Phe34, Gln38, Leu40, Leu41, Cys42, His57, Tyr60A, Leu65, Arg67, Arg73, Thr74, Tyr76, Glu80,	Lys36, Glu39, Lys60F, Met84, Gln151, Ile174, Asp189, Glu192	Pro37, Trp60D, Arg75, Lys81, Trp96, Glu97A, Asn143, Glu217, Gly219, Cys220, Tyr225, Phe227	Gln38, Leu40, Leu41, Cys42, Lys60F, Arg73, Thr74, Glu80, Gln151, Asp189, Gly193, Ser195, Ser214,	Arg73

	Ile82, Asn98, Leu99, Ala190, Cys191, Gly193, Ser195, Val213, Ser214, Trp215, Gly216, Glu217, Gly219, Gly226, Tyr228			Gly216, Gly219	
bza-2 hirulog (1qur)*	Met32, Phe34, Leu40, His57, Tyr60A, Leu65, Arg67, Arg73, Thr74, Tyr76, Ile82, Asn98, Leu99, Ala190, Cys191, Ser195, Val213, Ser214, Trp215, Gly216, Gly219, Gly226	Lys36, Pro37, Gln38, Glu39, Met84, Lys110, Asn143, Glu146, Ile174, Asp189, Glu192, Cys220, Tyr228	Trp60D, Lys60F, Arg75, Arg77A, Ser83, Trp96, Glu97A, Lys109, Thr147, Trp148, Lys149E, Gln151, Glu217, Arg221A, Tyr225, Phe227	Gln38, Leu40, Arg73, Thr74, Tyr76, Lys110, Lys149E, Asp189, Ser195, Gly216, Gly219	Arg73, Lys149E
Hirunorm IV (4thn)	Met32, Phe34, Gln38, His57, Tyr60A, Leu65,	Lys36, Leu40, Met84, Asp189, Glu192, Ser195,	Ser36A, Glu39, Trp60D, Lys60F, Arg75, Glu97A,	Arg73, Thr74, Arg75, Tyr76, Asp189, Ser214,	Arg73, Arg75, Asp189

	Arg67, Arg73, Thr74, Tyr76, Ile82, Asn98, Leu99, Ala190, Cys191, Trp215, Gly216, Gly219, Gly226, Tyr228	Val213, Ser214, Glu217, Cys220	Gln151, Ile174, Arg221A, Tyr225	Gly216, Gly219	
Hirunorm V (5gds)	Met32, Phe34, Gln38, His57, Tyr60A, Leu65, Arg67, Arg73, Thr74, Tyr76, Ile82, Asn98, Leu99, Ser195, Trp215, Gly216, Gly219, Cys220	Lys36, Leu40, Met84, Ile174, Cys191, Ser214, Glu217	Ser36A, Glu39, Trp60D, Lys60F, Arg75, Trp96, Glu97A, Glu146, Thr147, Gln151, Ala190, Glu192, Arg221A	Gln38, Arg73, Thr74, Ser195, Ser214, Gly216, Gly219	Arg73, Arg75
Hirutonin 2 (1ihs)*	Met32, Phe34, Gln38, Glu39, Leu40, Leu41, Tyr60A, Lys60F, Leu65, Arg67, Arg73,	Lys36, Cys42, His57, Cys58, Trp60D, Thr149, Asn149B, Val149C, Asp189, Glu192, Gly193,	Arg35, Pro37, Phe60H, Arg75, Ser83, Met84, Trp96, Glu97A, Asn143, Ile174, Glu217,	Gln38, Leu41, Lys60F, Arg73, Thr74, Tyr76, Ala149A, Asp189, Glu192, Gly193, Ser195,	Arg73

	Thr74, Tyr76, Ile82, Asn98, Leu99, Ala149A, Ala190, Cys191, Ser195, Val213, Trp215, Gly226, Tyr228	Ser214, Gly216, Gly219	Cys220, Arg221A, Tyr225, Phe227	Ser214, Gly216, Gly219	
Hirutonin 6 (1iht)*	Met32, Phe34, Cys42, Tyr60A, Arg67, Arg73, Thr74, Asn98, Leu99, Ala190, Cys191, Ser195, Val213, Trp215, Gly226	Gln38, Glu39, Leu40, Leu41, His57, Lys60F, Leu65, Tyr76, Ile82, Ile174, Asp189, Glu192, Gly193, Ser214, Gly216, Gly219, Tyr228	Trp60D, Arg75, Trp96, Glu97A, Glu217, Cys220, Tyr225, Phe227	Gln38, Leu40, Leu41, Arg73, Thr74, Asp189, Gly193, Ser195, Ser214, Gly216, Gly219	Arg73
Synthetic thrombin inhibitor P798 (1eoj)*	Met32, Phe34, Gln38, Leu40, Leu41, Cys42, His57, Cys58, Arg67, Arg73, Thr74, Tyr76, Ile82,	Glu39, Tyr60A, Leu65, Met84, Ile174, Glu192, Trp215, Gly219, Cys220, Tyr228	Trp60D, Lys60F, Phe60H, Arg75, Arg77A, Trp96, Glu97A, Gln151, Asp189, Glu217, Arg221A, Phe227	Leu41, Arg73, Thr74, Asp189, Gly216, Gly219	Arg73, Asp189, Ser195

	Asn98, Leu99, Ala190, Cys191, Gly193, Ser195, Val213, Ser214, Gly216, Gly226				
Synthetic thrombin inhibitor P596 (1hbt)	Met32, Phe34, Leu40, Cys42, His57, Tyr60A, Leu65, Arg67, Arg73, Thr74, Tyr76, Ile82, Asn98, Leu99, Ala190, Cys191, Glu192, Gly193, Ser195, Val213, Ser214, Trp215, Gly216, Gly226	Gln38, Glu39, Leu41, Met84, Asn143, Gln151, Asp189, Gly219, Tyr228	Lys36, Trp60D, Lys60F, Arg75, Arg77A, Lys81, Ser83, Trp96, Glu97A, Ile174, Glu217, Cys220, Tyr225, Phe227	Leu40, Arg73, Thr74, Gln151, Asp189, Cys191, Gly193, Ser195, Ser214, Trp215, Gly216, Gly219	-

* PDB entries that were manually curated for the analyses.

Table S4. Thrombin residues buried at different percentages (≥70%, 30-70%, 10-30%) upon complex formation with ligands bound at exosite II or at both exosite II and active site in the crystallographic structures of thrombin complexes. The protein residues involved in intermolecular hydrogen bonds (HB) and salt bridges (SB) are also reported. Functional partners, natural inhibitors, and synthetic compounds are highlighted in pink, yellow, and light blue, respectively. Analyses have been conducted using the PISA program [1].

Ligand (PDB entry)	≥ 70%	< 70% and > 30%	≤ 30% and ≥ 10%	HB	SB
**Exosite II**					
Fibrinogen γ′ peptide (2hwl)	His91, Ala129, Leu130, Ile162, Val163, Cys168, Phe181, Cys182, His230, Phe232, Arg233, Leu234	Arg93, Arg126, Arg165, Asn179, Lys235, Lys236, Trp237, Lys240	Pro92, Arg101, Asp125, Ala129A, Gln131, Ala132, Glu164, Asp178, Gln244	Arg93, Arg101, Arg126, Arg165, Asn179, Arg233, Lys235, Lys236, Lys240	Arg101, Arg233
Bovine kringle-2 (2hpp)	His91, Pro92, Arg93, Tyr94, Asn95, Arg101, Thr177, Trp237, Val241	Leu60, Ile90, Trp96, Arg97, Asp100, Arg175, Asp178, Lys236, Lys240, Gln244	Asn60G, Glu97A, Arg165, Ile176, Asn179, Arg233	Pro92, Arg93, Trp96, Arg97, Arg101, Arg175, Asp178, Lys240, Gln244	Arg93, Arg97, Arg101, Arg175, Lys236
Human kringle-2 (2hpq)	Pro92, Arg93, Tyr94, Asn95, Thr177, Trp237, Val241	Leu60, Ile90, His91, Trp96, Arg97, Arg101, Arg175, Asp178, Lys240	Tyr89, Glu97A, Asp100, Arg165, Ile176, Asn179, Lys236, Gln244, Phe245	Pro92, Arg93, Trp96, Arg97, Arg101, Asp178, Lys240	Arg93, Arg97, Arg101, Arg175, Lys236, Lys240

Heparin (1xmn)*	His91, Arg93, Trp237	Ala129A, Arg165, Phe232, Arg233	Pro92, Arg101, Arg126, Leu130, Asp178, His230, Leu234, Lys236, Lys240	His91, Arg233	Arg93, Arg101, Arg126, Arg165
Heparin (in the presence of antithrombin III) (1tb6)*	Arg93	His91, Pro92, Arg233, Lys236, Trp237, Arg101, Lys240	Asn179	-	Arg93, Arg233, Lys236
Platelet glycoprotein Ib*α* - GPIb*α* (1p8v)	His91, Glu127, Ser129B, Asn179, Phe232, Arg233, Leu234	Arg101, Arg126, Ala129A, Leu129C, Gln131, Phe204A, Lys235, Lys236, Trp237	Pro92, Arg93, Asp125, Tyr134, Asp178, Pro204, Asn204B, Gln239, Lys240	Arg101, Asn179, Arg233, Lys236, Lys240	Arg101, Glu127, Arg233
Toggle-25t/AF113-18 (5do4)*	His91, Arg101, Arg165, Asp178, Asn179, Arg233, Leu234, Trp237	Pro92, Arg93, Arg126, Ala129A, Gln131, Ala132, Glu164, Pro166, Lys169, His230, Phe232, Lys236, Lys240	Asp100, Leu130, Gly133, Val163, Arg175	Arg101, Gln131, Glu164, Arg165, Lys169, Asp178, Asn179, Arg233, Lys236, Trp237, Lys240	-
HD22_27mer (4i7y)	Tyr89, His91, Pro92, Arg93, Tyr94,	Trp96, Arg126, Val163, Asp178, Phe232,	Lys87, Ile88, Ile90, Arg97,	Pro92, Arg93, Asn95, Trp96, Arg97,	-

	Asn95, Arg101, Ala129, Leu130, Ile162, Arg165, Phe181, His230, Arg233, Trp237, Val241	Lys240, Phe245	Glu97A, Ala129A, Gln131, Ala132, Pro166, Lys169, Asn179, Met180, Leu234, Lys236, Gln244	Arg101, Arg126, Lys169, His230, Arg233, Trp237	
Suramin (2h9t)	His91, Trp237, Val241, Gln244	Pro92, Arg93, Lys240, Asp243, Gly246, Glu247	Tyr89, Arg101, Leu234, Lys236, Phe245	Arg101, Lys236, Glu247	-
**Exosite II + active site**					
Hemadin (1e0f)	His57, Tyr60A, Pro60C, Glu97A, Leu99, Arg173, Ile174, Ala190, Cys191, Glu192, Val213, Ser214, Trp215, Gly216, Glu217, Gly219, Cys220, Gly226	Trp60D, Arg97, Arg101, Glu146, Asp189, Ser195, Arg221A, Tyr228, Lys240	Lys60F, Pro92, Arg93, Trp96, Thr147, Arg175, Asp243, Gln244, Phe245	Trp60D, Arg93, Arg97, Glu97A, Arg101, Arg173, Asp189, Ala190, Ser195, Ser214, Gly216, Gly219	Arg93, Arg101, Asp189
Madanin-1 (5l6n)	His57, Tyr60A, His91, Arg93, Asn95, Glu97A, Asn98,	Trp60D, Tyr94, Arg97, Arg126, Asn179, Asp189,	Cys42, Pro92, Trp96, Asp125, Arg173, Arg175, Glu192,	His57, Tyr60A, Arg93, Arg97, Glu97A, Arg101, Arg126,	Arg101, Arg126, Asp189, Arg233

	Leu99, Arg101, Ile174, Ala190, Cys191, Ser195, Val213, Ser214, Trp215, Gly216, Gly226, Tyr228, Phe232, Arg233, Leu234, Trp237	Lys235, Lys236	Gly193, Glu217, Gly219, Cys220, Tyr225, Phe227, Lys240	Asn179, Asp189, Gly193, Ser195, Ser214, Gly219, Arg233, Lys236, Lys240	
Tsetse thrombin inhibitor (6tkl)	His57, Tyr60A, Arg93, Glu97A, Asn98, Leu99, Ala129, Leu130, Ala132, Ile162, Ile174, Phe181, Ala190, Cys191, Ser195, Val213, Trp215, Gly216, Gly219, Gly226, Tyr228, His230, Phe232, Arg233, Leu234	Pro60C, Trp60D, His91, Arg126, Gln131, Val163, Arg165, Asn179, Cys182, Ser214, Glu217, Cys220, Phe227, Lys235, Lys236, Trp237	Cys42, Lys60F, Pro92, Trp96, Arg97, Arg101, Asp125, Ala129A, Tyr134, Glu146, Glu164, Pro166, Arg173, Arg175, Asp178, Asp189, Glu192, Gly193, Arg221, Gln239, Lys240	His57, Tyr60A, Arg93, Glu97A, Arg101, Arg126, Leu130, Ala132, Arg165, Asn179, Asp189, Ala190, Gly216, Gly219, His230, Arg233, Lys235, Lys236	Arg93, Arg101, Asp189, Arg233, Lys236

* PDB entries that were manually curated for the analyses.

Table S5. Count of thrombin residues whose surface is buried at a percentage ≥70% upon complex formation with ligands bound at exosite I or at both exosite I and active site in the crystallographic structures of the selected thrombin complexes (46 PDB entries, see Table S3). Data are also reported by considering either non-aptameric (37 entries) or aptameric (9 entries) ligands. Residues interacting only with ligands bound to both exosite I and active site have not been considered.

Residue	All ligands	Non-aptamers	Aptamers
Tyr76	39	30	9
Arg67	35	34	1
Thr74	30	30	-
Leu65	29	27	2
Met32	28	28	-
Phe34	28	28	-
Ile82	26	26	-
Arg73	22	22	-
Gln38	18	18	-
Arg77A	13	5	8
Arg75	10	1	9
Glu77	9	-	9
Ile79	9	-	9
Lys36	5	5	-
Met84	4	4	-
Pro37	3	3	-
Trp148	3	3	-
Gln151	3	3	-
Ser36A	2	2	-
Glu80	2	2	-
Ser83	2	2	-
Glu146	2	2	-
Thr147	2	2	-
Val149C	2	2	-
Gly150	2	2	-
Lys145	1	1	-
Pro152	1	1	-
Ser153	1	1	-
Arg221	1	1	-

Table S6. Count of thrombin residues involved in intermolecular interactions (hydrogen bonds/salt bridges) upon complex formation with ligands bound at exosite I or at both exosite I and active site in the crystallographic structures of the selected thrombin complexes (46 PDB entries, see Table S3). Data are also reported by considering either non-aptameric (37 entries) or aptameric (9 entries) ligands. Residues interacting only with ligands bound to both exosite I and active site have not been considered.

Residue	All ligands	Non-aptamers	Aptamers
Thr74	34	31	3
Tyr76	32	23	9
Arg73	27	27	-
Arg77A	22	13	9
Arg75	20	11	9
Gln38	18	18	-
Leu40	11	11	-
Ile82	9	9	-
Glu77	8	-	8
Tyr117	8	-	8
Gln151	8	8	-
Asn78	6	-	6
Ser36A	5	5	-
Glu39	5	5	-
Lys81	5	5	-
Met84	5	5	-
Lys110	7	7	-
Trp184	4	4	-
Lys36	3	3	-
Met84	3	3	-
Lys145	2	2	-
Asp21	1	1	-
Asn62	1	1	-
Glu69	1	-	1
His71	1	-	1
Ser83	1	1	-
Pro111	1	1	-
Thr147	1	1	-
Gly150	1	1	-
Ser153	1	1	-

Table S7. Count of thrombin residues whose surface is buried at a percentage ≥70% upon complex formation with ligands bound at exosite II or at both exosite II and active site in the crystallographic structures of the selected thrombin complexes (12 PDB entries, see Table S4). Data are also reported by considering either non-aptameric (10 entries) or aptameric (2 entries) ligands. Residues interacting only with ligands bound to both exosite II and active site have not been considered.

Residue	All ligands	Non-aptamers	Aptamers
His91	8	6	2
Arg93	7	6	1
Trp237	7	5	2
Arg233	6	4	2
Leu234	5	4	1
Asn95	4	3	1
Arg101	4	2	2
Phe232	4	4	-
Val241	4	3	1
Pro92	3	2	1
Tyr94	3	2	1
Ala129	3	2	1
Leu130	3	2	1
Ile162	3	2	1
Phe181	3	2	1
His230	3	2	1
Arg165	2	-	2
Thr177	2	2	-
Asn179	2	1	1
Tyr89	1	-	1
Gly127	1	1	-
Ser129B	1	1	-
Val163	1	1	-
Cys168	1	1	-
Asp178	1	-	1
Cys182	1	1	-
Glu244	1	1	-

Table S8. Count of thrombin residues involved in intermolecular interactions (hydrogen bonds/salt bridges) upon complex formation with ligands bound at exosite II or at both exosite II and active site in the crystallographic structures of the selected thrombin complexes (12 PDB entries, see Table S4). Data are also reported by considering either non-aptameric (10 entries) or aptameric (2 entries) ligands. Residues interacting only with ligands bound to both exosite II and active site have not been considered.

Residue	All ligands	Non-aptamers	Aptamers
Arg101	11	9	2
Arg93	9	8	1
Lys236	9	8	1
Arg233	8	6	2
Lys240	6	5	1
Arg97	5	4	1
Arg126	5	4	1
Arg179	5	4	1
Arg165	4	3	1
Pro92	3	2	1
Trp96	3	2	1
Arg178	3	2	1
Lys169	2	-	2
His230	2	1	1
Lys235	2	2	-
Trp237	2	-	2
His91	1	1	-
Asn95	1	-	1
Glu127	1	1	-
Gln131	1	-	1
Glu164	1	-	1
Arg175	2	2	-
Gln244	1	1	-
Glu247	1	1	-

Table S9. Effect on the affinity of a ligand towards a thrombin exosite when another ligand is bound to the other exosite derived from experimental data.

**Exosite I ligand**	**Exosite II ligand**	**Effect on affinity towards:**		**References**
		**exosite I**	**exosite II**	
Hirudin	sF2[a]	Reduced	Reduced	[2,3]
Hirudin	Heparin	Reduced	ND	
Fibrin	γ'-peptide[b]	Reduced	ND	[4]
Fibrin	EV22[c]	Reduced	ND	
Fibrin	Heparin	Reduced	ND	
TM456[d]	GPIbα[e]	Reduced	ND	[5]
TM456[d]	γ'-peptide[b]	Reduced	ND	
TM456[d]	HD22_29mer	Reduced	ND	
PAR1 (49-62)[f]	(269-282, 3Yp)[g]	Increased	ND	[6]
PAR3 (44-56)[f]	(269-282, 3Yp)[g]	Increased	ND	
TBA	γ'-peptide[b]	ND	Reduced	[4]
Hirudin	γ'-peptide[b]	ND	Reduced	
TBA	HD22_38mer	Increased	Increased	[7,8]
TBA	HD22_29mer	Increased	Increased	

[a] Synthetic peptide corresponding to residues 63–116 of prothrombin fragment 2; [b] Analogue of the carboxy terminus of the γ'-chain of fibrinogen; [c] HD22 aptamer variant; [d] Thrombomodulin-derived peptide; [e] Peptide analogue of glycoprotein Ibα; [f] Protease activated receptors; [g] Triply phosphorylated GpIbα; ND: not determined.

References

1. Krissinel, E.; Henrick, K. Inference of Macromolecular Assemblies from Crystalline State. *J Mol Biol*, **2007**, *372*, 774–797.
2. Stone, S.R.; Hofsteenge, J. Effect of Heparin on the Interaction between Thrombin and Hirudin. *Eur. J. Biochem.*, **1987**, *169*, 373–376.
3. Fredenburgh, J.C.; Stafford, A.R.; Weitz, J.I. Evidence for Allosteric Linkage between Exosites 1 and 2 of Thrombin. *J. Biol. Chem.*, **1997**, *272*, 25493–25499.
4. Petrera, N.S.; Stafford, A.R.; Leslie, B.A.; Kretz, C.A.; Fredenburgh, J.C.; Weitz, J.I. Long Range Communication between Exosites 1 and 2 Modulates Thrombin Function. *J. Biol. Chem.*, **2009**, *284*, 25620–25629.
5. Chen, K.; Stafford, A.R.; Wu, C.; Yeh, C.H.; Kim, P.Y.; Fredenburgh, J.C.; Weitz, J.I. Exosite 2-Directed Ligands Attenuate Protein C Activation by the Thrombin-Thrombomodulin Complex. *Biochemistry*, **2017**, *56*, 3119–3128.
6. Billur, R.; Sabo, T.M.; Maurer, M.C. Thrombin Exosite Maturation and Ligand Binding at ABE II Help Stabilize PAR-Binding Competent Conformation at ABE I. *Biochemistry*, **2019**, *58*, 1048–1060.
7. Olmsted, I.R.; Xiao, Y.; Cho, M.; Csordas, A.T.; Sheehan, J.H.; Meiler, J.; Soh, H.T.; Bornhop, D.J. Measurement of Aptamer-Protein Interactions with Back-Scattering Interferometry. *Anal. Chem.*, **2011**, *83*, 8867–8870.
8. Feng, X.; Yu, C.; Feng, F.; Lu, P.; Chai, Y.; Li, Q.; Zhang, D.; Wang, X.; Yao, L. Direct Measurement of Through-Bond Effects in Molecular Multivalent Interactions. *Chemistry*, **2019**, *25*, 2978–2982.
